# Genomic and Phenomic Study of Mammary Pathogenic *Escherichia coli*

**DOI:** 10.1371/journal.pone.0136387

**Published:** 2015-09-01

**Authors:** Shlomo E. Blum, Elimelech D. Heller, Shlomo Sela, Daniel Elad, Nir Edery, Gabriel Leitner

**Affiliations:** 1 Department of Animal Sciences, Robert H. Smith Faculty of Agriculture, Food and Environment, Rehovot, Israel; 2 National Mastitis Reference Center, Department of Bacteriology, Kimron Veterinary Institute, Bet Dagan, Israel; 3 Microbial Food-Safety Research Unit, Department of Food Quality & Safety, Institute for Postharvest and Food Sciences, The Volcani Center, ARO, Bet Dagan, Israel; 4 Department of Bacteriology, Kimron Veterinary Institute, Bet Dagan, Israel; 5 Department of Pathology, Kimron Veterinary Institute, Bet Dagan, Israel; The Pennsylvania State University, UNITED STATES

## Abstract

*Escherichia coli* is a major etiological agent of intra-mammary infections (IMI) in cows, leading to acute mastitis and causing great economic losses in dairy production worldwide. Particular strains cause persistent IMI, leading to recurrent mastitis. Virulence factors of mammary pathogenic *E*. *coli* (MPEC) involved pathogenesis of mastitis as well as those differentiating strains causing acute or persistent mastitis are largely unknown. This study aimed to identify virulence markers in MPEC through whole genome and phenome comparative analysis. MPEC strains causing acute (VL2874 and P4) or persistent (VL2732) mastitis were compared to an environmental strain (K71) and to the genomes of strains representing different *E*. *coli* pathotypes. Intra-mammary challenge in mice confirmed experimentally that the strains studied here have different pathogenic potential, and that the environmental strain K71 is non-pathogenic in the mammary gland. Analysis of whole genome sequences and predicted proteomes revealed high similarity among MPEC, whereas MPEC significantly differed from the non-mammary pathogenic strain K71, and from *E*. *coli* genomes from other pathotypes. Functional features identified in MPEC genomes and lacking in the non-mammary pathogenic strain were associated with synthesis of lipopolysaccharide and other membrane antigens, ferric-dicitrate iron acquisition and sugars metabolism. Features associated with cytotoxicity or intra-cellular survival were found specifically in the genomes of strains from severe and acute (VL2874) or persistent (VL2732) mastitis, respectively. MPEC genomes were relatively similar to strain K-12, which was subsequently shown here to be possibly pathogenic in the mammary gland. Phenome analysis showed that the persistent MPEC was the most versatile in terms of nutrients metabolized and acute MPEC the least. Among phenotypes unique to MPEC compared to the non-mammary pathogenic strain were uric acid and D-serine metabolism. This study reveals virulence factors and phenotypic characteristics of MPEC that may play a role in pathogenesis of *E*. *coli* mastitis.

## Introduction

Mastitis, the inflammation of the mammary gland, is one of the most economically important diseases affecting dairy production [1,2]. Economic losses directly caused by mastitis in dairy farms include treatment expenses, lower milk yield and value, and culling of severely affected animals. Moreover, the whole dairy production chain is affected due to the delivery of low quality milk from sick animals, which impairs milk industrial processing [3,4]. Bovine mastitis is typically caused by infection of the mammary gland by pathogenic microorganisms present in the environment, skin or teat apex of dairy animals. *Escherichia coli* is one of the main pathogens causing bovine mastitis. While the prevalence of other important bovine mastitis pathogens, such as *Staphylococcus aureus* and *Streptococcus agalactiae*, was successfully reduced following massive mastitis control programs in various regions, *E*. *coli* remains a major etiological agent of bovine mastitis worldwide [5,6]. Upon the entry of *E*. *coli* into the mammary gland, mastitis onset is acute, developing to a local disease in the gland, and in extreme cases to a systemic disease. The time for clinical recovery and cessation of bacterial and increased somatic cells shedding in milk is generally considered to be short. However, in many cases, there are long term detrimental effects on mammary gland health and milk quality following an episode of *E*. *coli* mastitis, and in some cases the mammary gland does not fully recover [7]. Persistent *E*. *coli* infections in the mammary gland causing recurrent episodes of mastitis have long been documented [8]. Persistent infections may represent about 4.8% of clinical cases of *E*. *coli* mastitis [9], and are being increasingly recognized [5,7,10,11].

Pathogenic *E*. *coli* in humans and animals are often classified into pathotypes according to the presence of known mechanisms of virulence associated with a specific pathogenic process [12]. It has been proposed that mastitis causing *E*. *coli* may be considered as a new *E*. *coli* pathotype, for which the term “mammary pathogenic *E*. *coli*”, or MPEC, was suggested [13]. However, other than typical factors involved in *E*. *coli* virulence in general, evidence of the existence of specific pathogenic mechanisms attributed to this pathotype was not presented. A variety of *E*. *coli* compounds are known to be detected by the mammary gland epithelium and immune cells, subsequently triggering the local immune response that develops into mastitis. The most notorious of which is lipopolysaccharide (LPS) [14]. On the other hand, the active mechanisms employed by MPEC involved in the pathogenesis of mastitis remain largely unknown. *E*. *coli* bacteria isolated from intra-mammary infections lack most of the known virulence factors that are typically associated with pathogenicity in other forms of *E*. *coli* infections [15–20]. Yet, MPEC could be differentiated as a subset from *E*. *coli* strains randomly isolated from the environment by means of specific phenotypic properties associated with higher fitness in the mammary gland milieu, namely multiplication rate in milk and resistance to phagocytosis [21]. The phenotypic differences correlated to genotypic segregation assessed by pulsed-field gel electrophoresis (PFGE) of genomic DNA [21]. In addition, although a variety of PFGE genotypes may be found among MPEC [15,18,21,22], mastitis pathogenic strains were less genotypically diverse than environmental strains, as shown by multi-locus sequence typing [18], suggesting that *E*. *coli* strains causing mastitis are not random, and instead, may be under selective pressure based on the presence of virulence factors or fitness properties associated with the ability to infect the mammary gland and eventually leading to mastitis.

MPEC strains showing distinct virulence phenotypes *in vitro* are associated with different presentations of bovine mastitis. For instance, strains isolated from persistent IMI show higher ability to adhere and invade mammary epithelial cells than strains isolated from a single episode of transient mastitis [10,23]. Moreover, the bacterial internalization and intra-cellular trafficking mechanisms differ between these two types of strains [11]. Also, strains isolated from acute mastitis induced higher expression of chemokines and cytokines by mammary epithelial cells *in vitro* than a strain isolated from persistent, chronic mastitis [24]. However, the differences found *in vitro* in the virulence potential of *E*. *coli* associated with distinct mastitis presentations could not yet be clearly associated with genes encoding specific known virulence factors [18,20,24,25]. Thus, it is still questioned whether MPEC strains share a common set of pathogenicity factors that would distinguish them from other *E*. *coli* pathotypes and what are the specific pathogenic factors in MPEC leading to distinct mastitis presentations. In this context, whole genome sequence analysis would be a useful approach toward the identification of unique MPEC virulence factors associated with acute or persistent mastitis.

The objective of this study was to identify potential virulence properties in MPEC through whole genome and phenome comparative analysis. The genome and phenome of MPEC causing acute (strains VL2874 and P4) or persistent (strain VL2732) mastitis were compared to those of an environmental strain (strain K71), shown here to be non-pathogenic in the mammary gland. MPEC genomes were also compared to representative strains of different *E*. *coli* pathotypes.

## Materials and Methods

### Strains

Four *E*. *coli* strains were selected for this study. Three strains were originally isolated from bovine mastitis (P4, VL2874 and VL2732), and one strain was isolated from the environment in a cow shed (K71). Strain P4 was originally isolated from an acute case of mastitis and is widely used as a “model” strain in mastitis research [26]. The P4 isolate used in the current study was obtained directly from NCIMB (catalogue No. 702070), and was propagated only once before whole genome sequencing, thus representing the original strain described by Bramley *et al* [26]. The identity of this isolate was further confirmed by serotyping and phylogenetic group typing [18], and sequencing of its genome was previously published [27]. Strain VL2874 and genotypically identical isolates were found in a cohort of cases of highly severe mastitis that occurred in the same herd at relatively the same time [18]. Strain VL2732 was isolated from a case of persistent mastitis; genotypically identical bacteria were isolated from the same quarter of the mammary gland at different occasions during recurring episodes of mastitis for nearly seven months. Strains VL2874 and VL2732 were selected because they represent different presentations of mastitis, albeit they share a similar genotypic background [18]. Both strains caused long term detrimental effects on mammary gland health and milk quality, as previously described [7]. The fourth strain studied, K71, was isolated from a cow shed during a study comparing mastitis and non-mastitis strains [21]. This strain was assumed to be of low or no virulence in the mammary gland based on phenotypic characteristics that differed from phenotypes found in all mastitis strains in a previous study, namely slow multiplication rate in milk and low resistance to phagocytosis [21]. All the four strains were previously genotyped and studied for a wide range of known virulence factors [18]. After isolation, strains VL2732 and VL2874 were stored frozen in -80°C in brain heart infusion with 25% glycerol, whereas strain K71 was stored lyophilized in -18°C. The isolates used for whole genome sequencing in the current study corresponded to the second passage from the original isolates. Strains characteristics are summarized in Table 1. Published *E*. *coli* genomes used for intra-species genomic comparisons were selected to represented different *E*. *coli* pathotypes, namely avian pathogenic *E*. *coli* (APEC), uropathogenic *E*. *coli* (UPEC); neonatal meningitis *E*. *coli* (NMEC), enteropathogenic *E*. *coli* (EPEC), enterohemorrhagic *E*. *coli* (EHEC), adherent-invasive *E*. *coli* (AIEC), enteroaggregative *E*. *coli* (EAEC), enteroinvasive *E*. *coli* (EIEC), enterotoxigenic *E*. *coli* (ETEC) and *Shigella* spp. These are listed in detail in S3 Dataset.

**Table 1:** Characteristics of strains selected for whole genome sequencing in this study.

Strain	Source	Serotype^b^	Phylogenetic group^b^	ST^b^	PFGE cluster^b^	Growth in milk^b^/Resistance to phagocytosis^a^	Mammary pathogenic mice^c^/cows
VL2874	acute mastitis	O141:H4	A	10	II	fast/high	yes/yes
VL2732	persistent mastitis	O8:H30	A	10	II	fast/high	yes/yes
P4	acute mastitis	O32:H37	A	10	out group	fast/high	yes/yes
K71	cow shed	O58:H40	B1	58	out group	slow/low	no/no^d^

^a^ Blum et al., 2008

^b^ Blum et al., 2013

^c^ Present study

^d^ Blum et al., in press

### Intra-mammary pathogenicity

Pathogenicity in the mammary gland (or lack thereof, for strain K71) was evaluated *in vivo* in a model of intra-mammary challenge in female mice as previously described [28], with modifications as follows. Briefly, bacteria were grown to log phase in nutrient broth (Merck, Darmstadt, Germany) at 37°C and washed in non-pyrogenic phosphate-buffered saline (PBS). An aliquot was serially diluted and plated on blood agar plates (Tryptose Blood Agar Base; Becton-Dickinson, Sparks, MD, USA, enriched with 5% washes sheep red cells) for colony forming units (CFU) counting. An average of 5x10^2^ CFU (range 4-7x10^3^ CFU) in 5 μL were injected subcutaneously into the left abdominal mammary gland (L4) of Swiss female mice 7–10 days post-partum using a 30-G needle, with care not to injure blood vessels. This route of injection was chosen to avoid injuring the teat, which could lead to unspecific reaction in the gland. Mice were euthanized one, two and five days post-challenge (DPC). The experiment was performed in triplicates. Injected glands were observed for gross pathology externally and internally in comparison to the contralateral mammary gland, then removed, weighted and examined by bacterial culture and histopathology. Animal experiments were approved by the Kimron Veterinary Institute Committee of Animal Experimentation.

### Growth in milk and nutrient broth

Growth rates were tested in pasteurized whole milk and in nutrient broth as previously described [21]. Briefly, bacteria were inoculated into 10 ml of either milk or nutrient broth and incubated in normal atmosphere at 37°C. Bacterial growth was measured as CFU/ml by plate counts after 4 and 8 h. This test was performed in triplicates for each strain and mean bacterial concentration of each strain at each time point was compared by t-test.

### Phenome analysis

The phenome of each strain was assessed by Phenotype MicroArrays (BiOLOG, Hayward, CA). Metabolic utilization of a total of 758 nutrient substrates was measured at the company’s laboratories following the manufacturer's instructions [29], and included plates PM1, PM2A, PM3B, PM4A and PM5 through 8, comprising 190 carbon, 95 nitrogen, 59 phosphorous, 35 sulfur and 285 peptide nitrogen sources, and 94 nutrient stimulants. Nutrients utilization was measured in duplicates for 24 h in 15 min intervals. Data analysis and visualization was made in R [30] using the OPM package version 1.1.0 [31,32]. The area under the curve (AUC) was extracted, and reactions were discretized into positive, weak or negative using the k-means method [32]. K-means discretization was performed including data of all strains and duplicates for each plate type (PM1 to 8) separately. In plate PM4, phosphorous and sulfur reactions were analyzed independently. Discrepancy between replicates was resolved as follows: reactions with one positive and one weak duplicate were considered positive, whereas reactions with one negative and one weak duplicate were considered negative. Negative and positive controls of each PM plate were not included in the total number of reactions.

### DNA extraction, genome sequencing and assembling

Bacteria were grown overnight at 37°C on blood agar (Tryptose Blood Agar Base, Becton-Dickinson, Sparks, MD, USA, enriched with 5% washed sheep red blood cells). Bacterial DNA was extracted using MagNa Pure Compact Nucleic Acid Isolation Kit I and libraries were prepared with GS FLX Titanium Rapid Library Preparation Kit (Roche Applied science, Mannheim, Germany). Genomes were sequenced using Roche 454 GS FLEX Titanium technology (Dynlabs, Zerifin, Israel). Sequences were assembled *de novo* using Newbler 2.6. The presence of plasmids was assessed by plasmid DNA extraction with QIAprep Miniprep (QIAGEN) for small plasmids, and a heat lysis method previously described [33] to include large plasmids. Briefly, overnight cultures of *E*. *coli* were pelleted and lysed in 3% SDS and 50 mM Tris (pH 12.6) at 55°C for one hour, followed by phenol-chloroform DNA extraction. Plasmids were visualized with gel electrophoresis.

### Genome annotation and analysis

*De novo* assembled contigs were reordered using MAUVE [34]. The genome of *E*. *coli* strain K-12 MG1655 (NC_000913) was used as a reference for strains P4, VL2732 and VL2874 due to their phylogenetic proximity (phylogenetic group A, ST10). For strain K71, phylogenetic group B1 strain IAI1 (NC_011741) was chosen as a reference instead. Reordered contigs were concatenated and genomes were aligned using ACT [35] and inspected in ARTEMIS [36]. Annotation and functional comparisons were performed using the RAST server [37]. Plasmid sequences were identified based on a combination of annotation features, megablast [38] and using PlasmidFinder [39], and corroborated by plasmid DNA visualization as described above.

### Single nucleotide polymorphism based phylogenetic analysis

Single nucleotide polymorphisms (SNP) on whole genome sequences were identified using the snpTree 1.1 server [40] with the genome of strain K-12 MG1655 as reference. Concatenated SNPs were aligned online and used to generate a phylogenetic tree with FastTree 2.1.5 using the Generalized Time-Reversible model [41]. Genomes for comparison were selected as to represent different *E*. *coli* pathotypes and genealogies. These are listed in detail in the S3 Dataset.

### Genome-to-Genome Distance (GGD)

Genome-to-genome distances were calculated by the Genome Blast Distance Phylogeny approach [42] using the Genome-to-Genome Distance Calculator 2.0 online (http://ggdc.dsmz.de/) set to BLAST+. Distance values obtained from formula 2 were used to build a heatmap of distances between each of the four studied genomes and selected published *E*. *coli* genomes representing several *E*. *coli* pathotypes presented in detail in the S3 Dataset. The genome of *E*. *fergusonii* ATCC 35469 (NC_011740, NC_011743) was used as out group in GGD analysis.

### Proteome comparison

Genomes were compared at the proteome level using CMG-Biotools [43] using a subset of the genomes listed in the S3 Dataset. For consistency across annotation, proteome files in FASTA format were downloaded from the PATRIC database [44], where all genomes are annotated using RAST. Proteomes were compared pairwise by all-against-all BLAST [45], and genes were clustered into families. A modified threshold of 80% sequence homology over 80% of sequence alignment length was used to define two proteins as belonging to the same gene family. A dendrogram representing the relative Manhattan distance between proteomes on basis of gene presence/absence was generated from the calculated pan-genome for the studied set of proteomes considering all genes, including singletons, i.e. genes present in a single genome ("flat" method in CMG-Biotools). A similarity matrix was built using the proportion of shared gene families between every two genomes, with homologous hits within the same proteome depicted at the bottom row of the matrix. The following subsets of genes were selected for further analysis: a. the intersection of MPEC proteomes complementary to (not found in) strain K71, b. genes specific for each MPEC proteome, and c. the intersection of MPEC proteomes complementary to all other genomes used in the comparison. These proteins were identified using the RAST annotation of each genome. In addition, BLAST2GO [46] was used in an attempt to improve annotation of these proteins.

## Results and Discussion

### Intra-mammary pathogenicity

**Fig 1:**
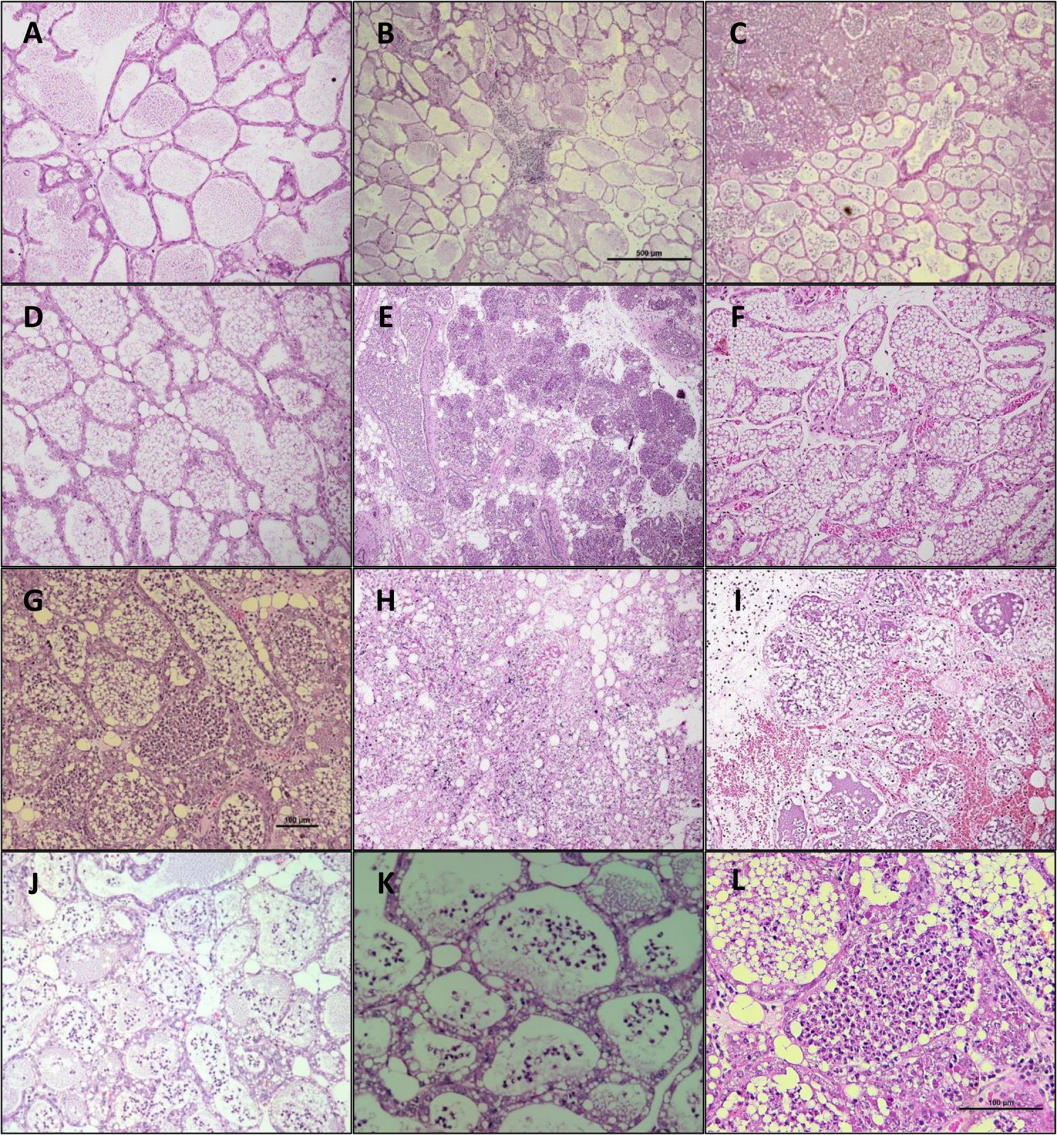
Histological examination of mice mammary glands. A, non-pyrogenic phosphate-buffered saline (negative control), 1 DPC, no histological changes (x10); B, strain K71, 1 DPC, focal light inter-alveolar infiltrate with no signs of inflammation of the gland (x4); C, strain P4, 1 DPC, focal intra-alveolar PMN infiltration (x4); D, strain P4, 2 DPC, recovery of the gland and no inflammatory infiltrate or significant damage (x10); E, strain VL2874, 1 DPC, extensive intra-alveolar and ductal PMN infiltration (x4); F, strain VL2874, 2 DPC, partially destructed non-lactating alveoli, protein deposit and necrosis (x10); G, strain VL2732, 1 DPC, diffuse inter and intra-alveolar PMN infiltration (x10); H, strain VL2732, 2 DPC, inter-alveolar inflammatory infiltrate, necrosis, hyperemia and loss of alveoli architecture (x10); I, strain VL2732, 5 DPC, degenerative PMN, diffuse necrosis, hemorrhage, protein deposit and extensive loss of alveoli architecture (x10); J-K, strain K-12 MG1655, 1 DPC, diffuse intra-alveolar PMN infiltration without alterations to the gland structure and numerous lactating alveoli (x4-x20); L, representative picture of intra-alveolar PMN infiltration (x20). Eosine-hematoxylin stain.

Pathogenicity in the mammary gland was studied in a murine model of intra-mammary infection. The murine model of intra-mammary infection has been widely used for the research of pathogenesis, immune response, treatment and prevention of mastitis, but only in a few instances it was used for comparison between the pathogenicity of different strains of the same pathogen species [47]. As expected, all three strains isolated from mastitis elicited clear intra-mammary inflammation in mice. Moreover, different patterns of inflammation were observed with the distinct MPEC strains on gross and histopathological examination of challenged mammary glands, and an analogy could be made between these patterns and the disease presentation observed in the mammary glands of cows originally infected by the strains studied here. For instance, strain VL2874 caused fast degeneration of mice glands within 1 DPC with extensive tissue destruction, whereas strain VL2732 caused a prolonged inflammation up to 5 DPC with a granuloma-like reaction, indicative of a chronic reaction. Comparably, the cow affected by VL2874 showed per-acute mastitis, no recovery of the affected gland to lactation, and extensive regions of gland tissue degeneration were observed histologically after culling. In contrast, VL2732 caused persistent, chronic infection in the cow mammary gland from which it was originally isolated. Compared to the two previous strains, strain P4 caused a milder inflammation in murine mammary glands. Results are summarized in Table 2 and representative histopathological images are presented in Fig 1. In accord to previous work [48], the murine model was shown to be applicable for the purpose of MPEC inter-strain comparisons. Finally, no signs of mastitis whatsoever were observed in mice challenged with strain K71, confirming that strain K71 was suitable for comparison to MPEC for the identification of potential mastitis pathogenicity-related traits.

**Table 2:** Gross and histopathological examinations of mammary glands injected by three mammary pathogenic and one non-pathogenic, environmental strain.

Strain	DPC	Gross pathology changes		Histopathological changes		
		External	Internal	PMN infiltrate	Mammary tissue	Mammary lymph node
VL2874	1	complete atrophy	complete atrophy, hyperemia	intra-alveolar, extensive	none	reactive
	2	complete atrophy	complete atrophy, hyperemia	none	non-lactating alveoli, partial destruction of alveoli	reactive
VL2732	1	none	none	diffuse, inter and intra-alveolar	none	partially reactive
	2	massive swelling	strong hyperemia, crepitation, swelling, hardness, granuloma-like	inter-alveolar	necrosis, hyperemia, loss of alveoli architecture	reactive
	5	massive swelling	swelling, hardness	degenerate	diffuse necrosis, loss of alveoli architecture	reactive, PMN infiltrate, capsular necrosis
P4	1	none	light hyperemia	intra-alveolar, focal	none	reactive
	2	partial atrophy	light hyperemia, partial atrophy	none	none	reactive
K71	1	none	light focal hyperemia	inter-alveolar, few	none	None
	2	partial atrophy	partial atrophy	inter-alveolar, few	none	None

PMN, polymorphnuclear cells.

### Phenome analysis

**Fig 2:**
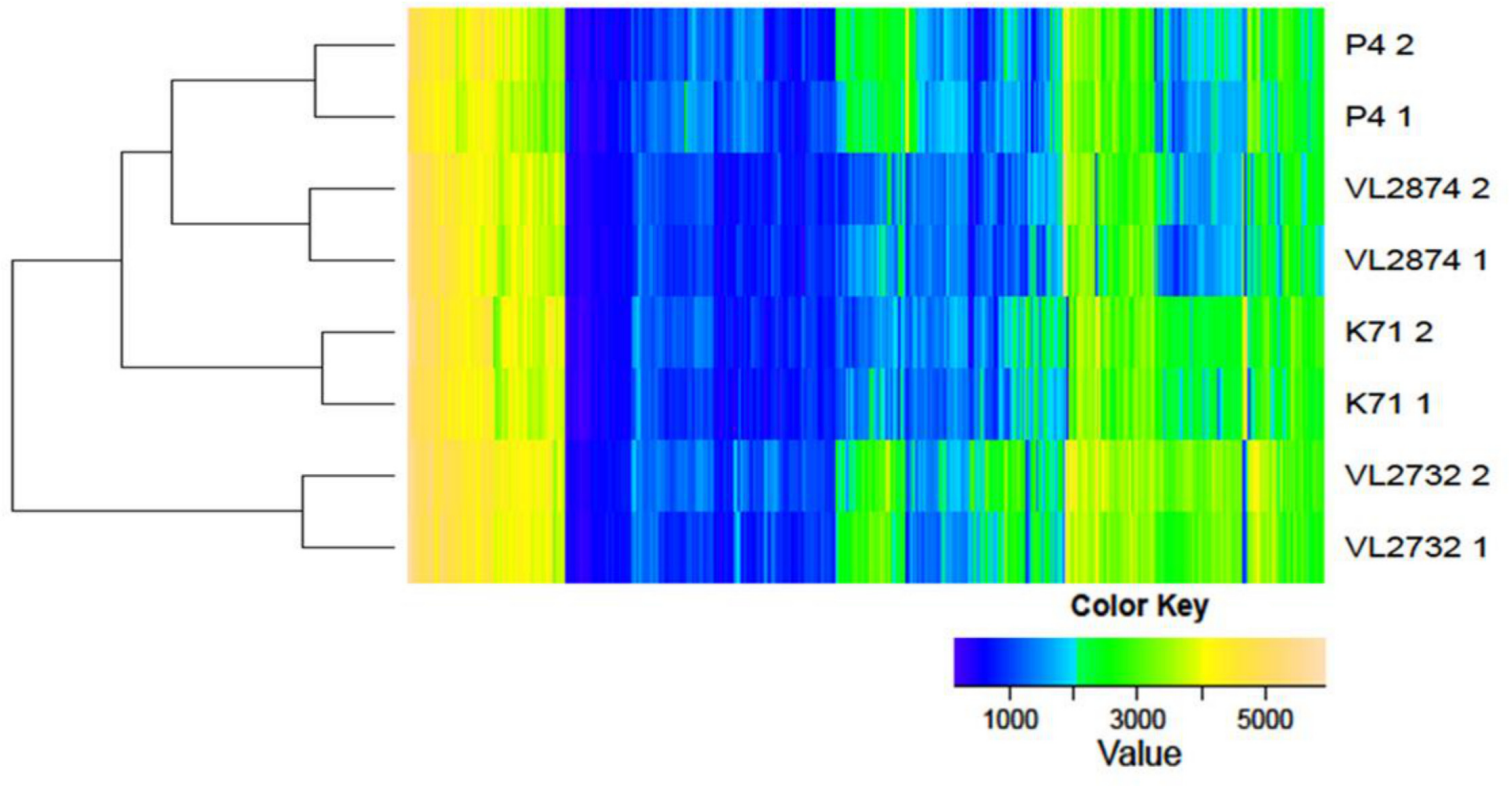
Heatmap of the area under the curve of phenotype microarray. Heatmap of the area under the curve (AUC) parameter extracted from kinetic data over 24 h in phenotype microarray with validation by 100 bootstrap repetitions. Numerals after the strain name indicate technical replicates of the same strain. The heatmap color indicates the AUC; yellow for higher values and blue for lower.

The metabolic utilization of a total of 758 nutrient sources was assessed. Reactions were categorized into positive, weak or negative based on AUC values. All four strains were negative to a total of 243 reactions, weak to 31 reactions and positive to 127 reactions. A heatmap of AUC values for all reactions is presented in Fig 2, showing that strains P4 and VL2874 were similar, and strain VL2732 was the most divergent one. The number and percentage of positive reactions by nutrient type are presented in Table 3. Strain VL2732 was the most versatile in terms of the number of nutrients metabolized. Accordingly, the number of unique positive reactions in each strain, i.e. positive reactions in a single strain that were negative or weak in the other ones, was higher for strain VL2732 (n = 123, representing 36% of the positive reactions in this strain), in contrast to K71 (n = 7, 4% of positive reactions in this strain), VL2874 (n = 3, 2% of positive reactions in this strain) and P4 (n = 5, 3% of positive reactions in this strain). Notably, strain VL2732 showed the highest versatility in the ability to use peptides as a source of nitrogen, to metabolize organic sulfur compounds and in the responsiveness to nutritional supplements (measured as improved growth over non-supplemented medium). Whether these characteristics provide this strain with an advantage to explore the intra-cellular niche, thus allowing it to install a persistent infection in the mammary gland, could be a subject of further investigation.

**Table 3:** Positive reactions for utilization of nutrient sources by category. In brackets, the percentage of positive reactions in a particular nutrient category is shown.

Nutrient type (no. of reactions)	VL2874 (%)	VL2732 (%)	P4 (%)	K71 (%)
carbon (190)	41 (22)	46 (24)	41 (22)	48 (25)
nitrogen (95)	18 (19)	21 (22)	16 (17)	14 (15)
nitrogen peptides (285)	79 (28)	127 (45)	75 (26)	82 (29)
phosphorous (59)	26 (44)	44 (75)	24 (41)	35 (59)
sulfur (35)	5 (14)	19 (54)	5 (14)	8 (23)
nutrient stimulation (94)	1 (1)	81 (86)	3 (3)	4 (4)
Total (758)	170 (22)	338 (45)	164 (22)	191 (25)

Ten reactions were found for which the three MPEC strains were positive whereas the non-mammary pathogenic strain K71 was either negative or weak. These were six peptide and two amino acid nitrogen sources, one organic phosphorous source and one amino acid carbon source. One of the two amino acids metabolized only by MPEC was D-serine, either as carbon or nitrogen source. D-serine may be bacteriostatic for *E*. *coli*, and the inability to metabolize D-serine affects growth and virulence, as shown in UPEC and NMEC [49]. Whether similar effects would influence the pathogenicity of MPEC in the mammary gland or not should be further studied. The non-mammary pathogenic strain K71 was able to metabolize sucrose, whereas none of the three MPEC strains were able to do so. Interestingly, although *E*. *coli* strains infecting the mammary gland are regarded to be essentially of fecal origin, the differences in these two phenotypes (D-serine and sucrose) are in accord to studies showing that the ability to metabolize D-serine is actually associated with extra-intestinal *E*. *coli*, whereas the ability to metabolize sucrose instead is associated with intestinal strains [49,50]. The inability of strain K71 to metabolize D-serine can be attributed to the absence of the *dsdC* gene for D-serine dehydratase (*dsdA*) transcriptional activator, and the insertion of the sucrose-6-phosphate hydrolase (EC 3.2.1.26) and the sucrose specific transcriptional regulator CscR upstream to the D-serine *dsd* operon, as found by genomic analysis. A similar impairment of the D-serine operon by insertion of a sucrose operon was described in various intestinal *E*. *coli* strains [49].

In addition, the three mastitis pathogenic strains were able to metabolize uric acid as a nitrogen source. Although the reaction was weak, it was completely negative in strain K71. Uric acid was found to have a role in the oxidative antimicrobial defenses of milk in the mammary alveoli during mastitis [51]. Thus, it is possible that the ability to metabolize uric acid is an advantageous phenotype of MPEC during intra-mammary infection. A full list of results of the PM assay is provided in S1 Dataset (raw data available upon request).

One of the characteristics previously described differentiating MPEC and environmental *E*. *coli* strains was growth rate in milk, which was correlated to assimilation of lactose [21]. In that study, strain K71 showed low levels of lactose fermentation. However, no differences in lactose metabolism were found between MPEC and strain K71 in the phenotype microarray here. It should be noted, however, that the method used previously and the phenotype microarray used here measure lactose metabolism in different ways (previously, lactose assimilation was measured by means of acidification of the medium, here by bacterial respiratory activity). Furthermore, no differences were found in the lactose operon sequences between MPEC and K71, thus suggesting that other metabolic pathways may be involved in the phenotypic differences reported for growth in milk.

### Genome analysis

Average sequencing reads counts ranged from 280,000 to 336,000 reads. Reads length ranged from 291 to 230 bases. *De novo* assembling metrics and basic annotation results are described in S1 Table and S2 Table, respectively. All the four genomes were of comparable size, gene contents and gene density. Strain K71 showed the largest genome and gene number.

**Fig 3:**
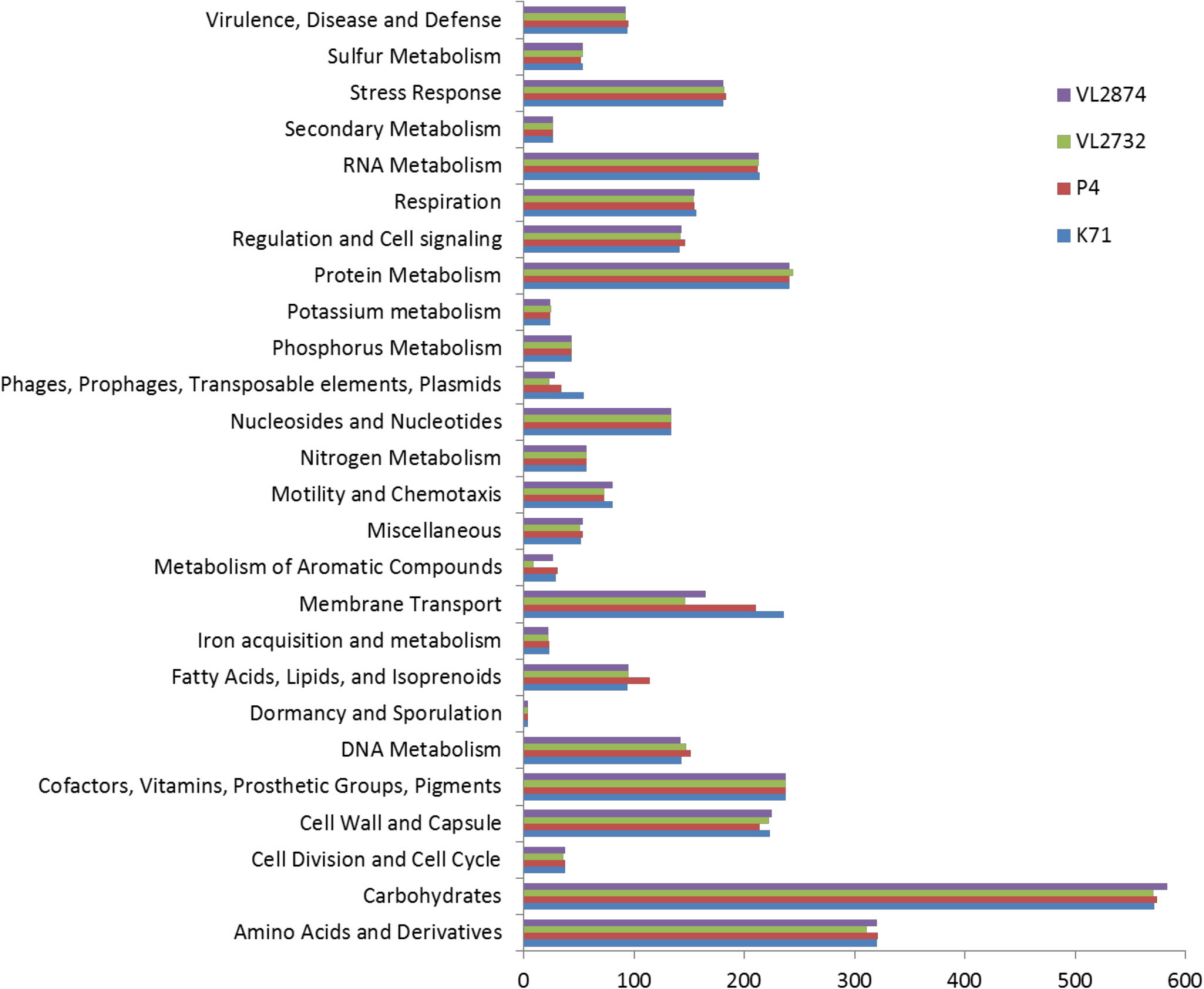
Distribution of functional annotation into RAST subsystems.

Overall, the four genomes showed a similar distribution of features into subsystem categories using the RAST subsystems annotation (Fig 3). Differences were found notably in mobile elements (phages, plasmids), metabolism of aromatic compounds, membrane transport and lipid metabolism categories. Many elements belonging to metabolism of aromatic compounds were lacking in strain VL2732. Differences in the membrane transport subsystem were attributed to features belonging to type IV secretion system found in strains K71 and P4, and to type VI secretion system found in strains K71 and VL2874. Differences in the lipid metabolism subsystem were attributed to a phospholipid and fatty acid biosynthesis related cluster that is present only in strain P4.

Differential comparison revealed 165 functional features in common in the three MPEC strains that were absent in strain K71. Among these, notable functions were: a. carbohydrate utilization (galactosamine utilization, sugar kinase cluster Ygc and carbohydrate utilization cluster Ydj), b. membrane antigen (biosynthesis of lipopolysaccharide, enterobacterial common antigen and outer membrane lipoprotein), c. membrane transport (beta-fimbriae, protein secretion system type VII) and d. iron uptake (iron(III)-dicitrate system). From the above, lipopolysaccharide (LPS) is considered a major virulence factor in *E*. *coli* mastitis [6]. Intra-mammary injection of purified LPS alone is able to trigger a local inflammatory reaction in the mammary gland that resembles, although is not identical to actual *E*. *coli* infection [52]. LPS biosynthesis, specifically of the core oligosaccharide region, seem to be impaired in strain K71 due to the lack of functional enzymes LPS 1,2-N-acetylglucosaminetransferase (EC 2.4.1.56), LPS 1,6-galactosyltransferase (EC 2.4.1.-) and the LPS core biosynthesis proteins RfaS and RfaZ. It is possible therefore that impaired LPS biosynthesis together with altered biosynthesis of additional membrane antigens, such as the enterobacterial common antigen (ECA) and the outer membrane (OM) lipoprotein, would affect immunogenicity and attenuate or prevent an inflammatory response against this strain. Another important feature lacking in strain K71 is the iron(III)-dicitrate system for iron uptake. In fact, this system for iron acquisition was previously found to be relatively prevalent in *E*. *coli* bacteria isolated from bovine mastitis [53]. The system is induced by ferric dicitrate, which is actually the main iron-chelating mechanism found in milk during lactation. The lack of this effective iron acquisition system in milk, in spite of the presence of other, perhaps less relevant systems, could limit bacterial growth of strain K71inside a lactating mammary gland, consequently limiting its ability to establish an intra-mammary infection. The specific features found in common in MPEC and lacking in strain K71 and the expected effects on virulence are summarized in Table 4. The full functional RAST annotation of MPEC genomes for comparison is provided in S2 Dataset.

**Table 4:** Main functional features present in the three MPEC strains and lacking in the non-mammary pathogenic strain K71 and their expected effects on virulence.

system	function	expected effect on
carbohydrate utilization	galactosamine utilization (PTS system, galactosamine-specific IIB component)	growth
	sugar kinase cluster Ygc	growth
	carbohydrate utilization cluster Ydj	growth
membrane antigen	LPS	immunogenicity, resistance (cell wall stability)
	ECA (lipid III flippase WzxE)	immunogenicity, resistance (cell wall stability)
	OM lipoprotein SmpA	immunogenicity, resistance (cell wall stability)
membrane transport	beta-fimbriae	adhesion
iron uptake	iron(III)-dicitrate system	growth
amino acids	D-serine dehydratase transcriptional activator	resistance

**Fig 4:**
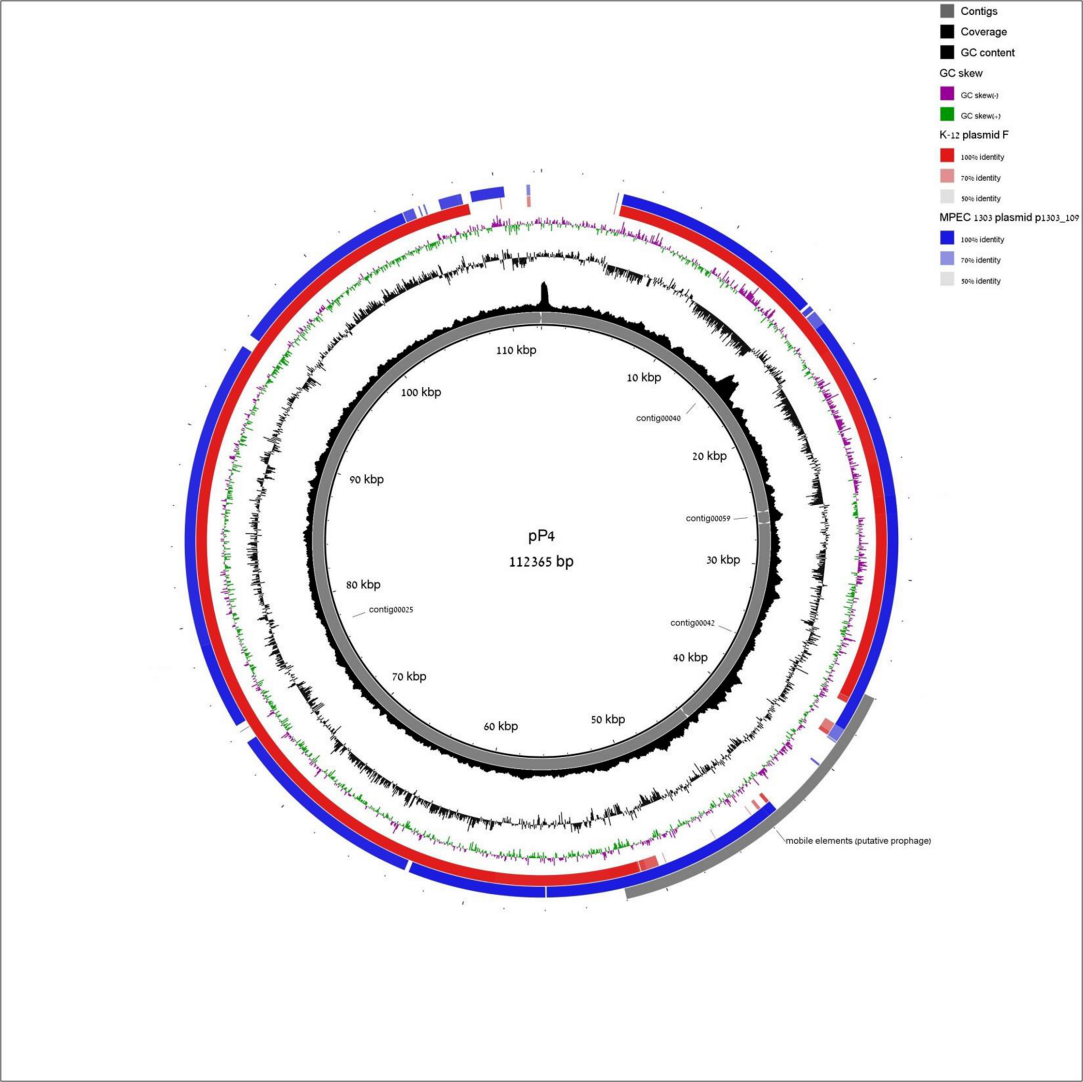
Plasmid pP4 of strain P4. Alignment of predicted sequence of conjugative plasmid pP4 of strain P4 aligned to plasmid F of strain K-12 and to plasmid p1303_109 of mastitis strain 1303.

Strain specific features were found in each of the three MPEC strains studied (50, 91, 130 functions in strain VL2874, VL2732 and P4, respectively). Strain P4 specific features were mainly associated with prophages and with phospholipid and fatty acid biosynthesis. As previously reported [27], strain P4 also harbors a conjugative plasmid type IncF IC(FII), here named pP4, comprising contigs 25, 40, 42 and 59 of the current assembly. The presence of this plasmid in the genome of strain P4 was confirmed here by gel electrophoresis (data not shown). *In silico* analysis showed that the pP4 plasmid is estimated to be about 112 Kb long, to have a 47.7 GC percentage and to include 118 CDS. Plasmid pP4 is nearly identical to the plasmid F of strain K-12 (AP001918). Although virulence factors were not detected on the pP4 plasmid, an interesting observation was the presence of a region of 14,639 bp beyond the K-12 plasmid F backbone. BLAST search of this region revealed that it is 99% identical to plasmid p1303_109 (CP009167), found in the mammary pathogenic *E*. *coli* strain 1303 that was also isolated from acute bovine mastitis [54] (Fig 4). This region has 45.7 GC percent and includes 16 CDS (11 mobile element proteins, 3 hypothetical proteins and co-activators of prophage gene expression IbrA and IbrB).

In strains VL2874 and VL2732, specific features were found that could be related with increased virulence or potential for persistence in the mammary gland.

A notable feature found in strain VL2874 was a RTX toxin cluster, including the hemolysin genes *hlyA*, *hylC* and *hylD*. Indeed, strain VL2874 is the only hemolytic strain on sheep-blood agar out of the four strains studied here. HlyA was shown to enhance the pathogenicity of extra-intestinal *E*. *coli* (ExPEC). A similar RTX toxin, TosA, was implicated in uropathogenic *E*. *coli* (UPEC) pathogenicity [55]. It is possible therefore that the RTX cluster found in the highly virulent strain VL2874 is associated with cytotoxic properties in the mammary gland, which would lead to tissue injure and consequently to per-acute mastitis, as observed in cows infected by this strain. The RTX cluster in strain VL2874 was found in a region of plasmid origin, along with F4-like fimbriae, which could be involved in adherence to mammary epithelial cells. *In silico* analysis indicated that this is a large, about 98 Kp long, type FII plasmid; although the whole sequence of this plasmid is yet to be finished. A megablast search for similar plasmids in the complete *E*. *coli* plasmids database in NCBI revealed the following plasmids covering about 43% of the large plasmid in strain VL2874, pVir68 (NC_012944, bovine septicemia), p1303_109 (NZ_CP009167, mastitis), pECC-1470_100 (NZ_CP010345, mastitis) and 39%, K-12 plasmid F (NC_002483). A second plasmid identified in the genome of strain VL2874 (contig 84) is a small plasmid type Col(RNAI) that is highly similar to the ColE-1-like plasmid p302S (AY333433) found in *Salmonella enterica* subsp. *enterica* [56] and that confers resistance to kanamycin. Although the small plasmid of stain VL2874 is larger than p302S, it lacks the aminoglycoside 3'-phosphotransferase type 1 gene. Similar small plasmids are also found in various *E*. *coli* strains, but with lower identity to the small plasmid of strain VL2874. Both plasmids of strain VL2874 were confirmed by gel electrophoresis (data not shown).

**Fig 5:**
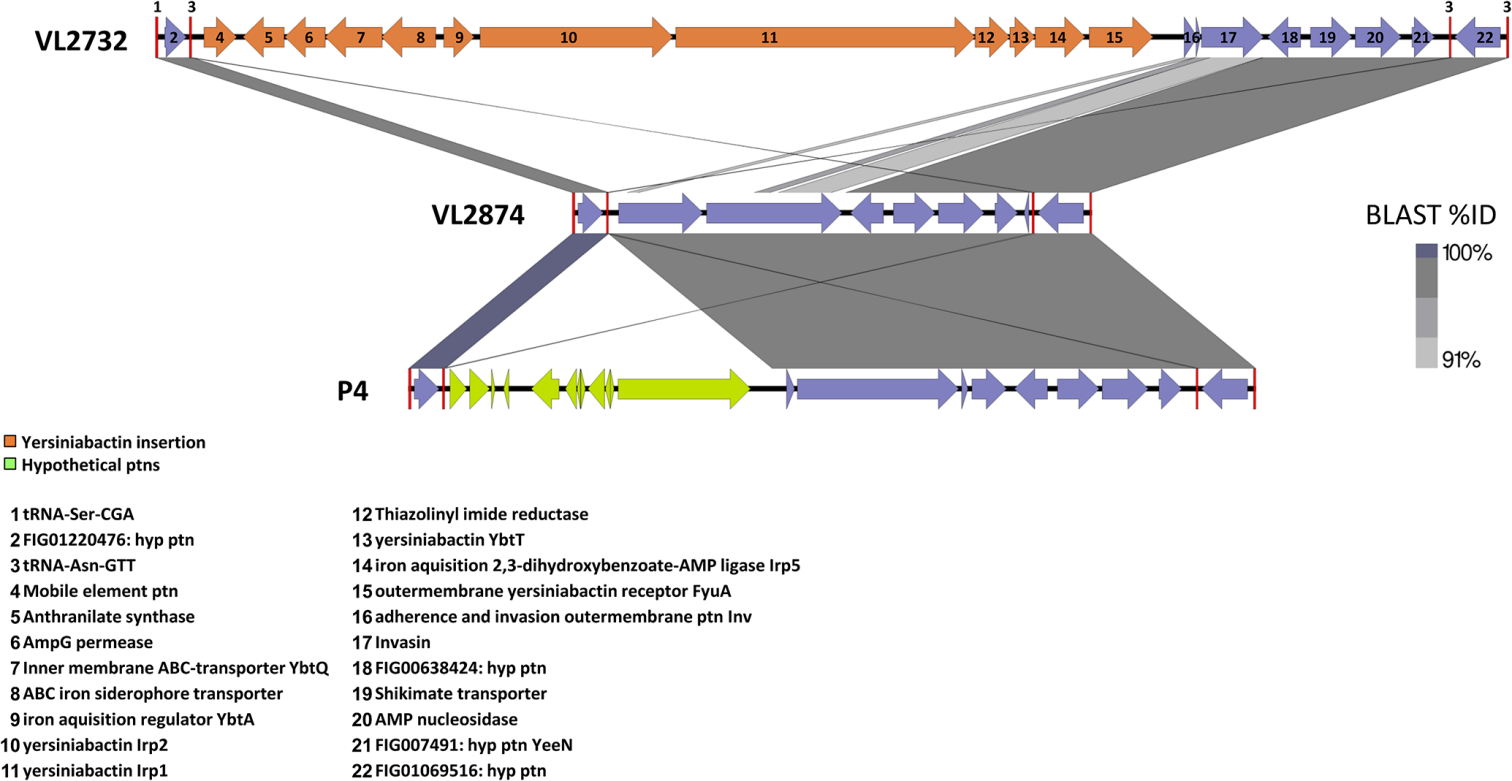
VL2732 Pathogenicity Island containing the yersiniabactin and invasin Inv clusters, resembling the Yersinia high pathogenicity island. The figure shows the alignment of the PAI region in the genome of strain VL2732 and the genomes of MPEC strains VL2874 and P4, showing the insertion site of the yersiniabactin elements.

The specific features found in strain VL2732 compared to the other MPEC studied here included the toxin mRNA interferase YgiU, or MqsR (motility quorum sensing regulator), which regulates biofilm formation and shift into persistent (dormant) cells under stress [57], and the yersiniabactin iron acquisition system, which is also associated with biofilm formation in iron limited environments, such as milk [58], and which may support intracellular survival through copper binding [59]. The presence of yersiniabactin was previously reported in another *E*. *coli* strain isolated from persistent mastitis [20], reinforcing a possible role of this system in the pathogenesis of persistent *E*. *coli* mastitis. Yersiniabactin was shown to be a critical iron acquisition system in another extra-intestinal *E*. *coli* pathotype, APEC. In fact, yersiniabactin and other iron acquisition systems, namely aerobactin, enterobactin and iron(III)-dicitrate, are not functionally redundant, as the deletion of yersiniabactin impairs growth in iron-limited medium even in the presence of the other systems [60]. It is possible therefore that yersiniabactin may have a role in promoting prolonged survival of persistent strains in the mammary gland, even though aerobactin, enterobactin and iron(III)-dicitrate were all found in the three MPEC strains studied here. The yersiniabactin cluster was found in strain VL2732 in a pathogenicity island (PAI) between tRNA-Asn-GTT (contig 1, 336,922:336,994), flanked by mobile elements, and tRNA-Asn-GTT (376,893:376,821) (Fig 5). This PAI also comprises the invasin Inv cluster, resembling the *Yersinia* high pathogenicity island (HPI) [61]. Strain VL2732 also bears the manganese ABC transporter SitABCD, which besides being a further iron acquisition system, also protects bacteria from oxidative stress [62], and could thus support intra-cellular survival. Overall, the VL2732 specific characteristics may promote the ability of this strain to cause persistent infections in the mammary gland [10,63]. Contrary to the other two MPEC strains above, no plasmids were detected in the genome of strain VL2732, either *in silico* or by gel electrophoresis (data not shown).

Interestingly, strain K71, albeit non-pathogenic in the mammary gland, is not completely devoid of factors that could be associated with virulence in other sites. For instance, features found in strain K71 that could be related to virulence were type VI secretion system, alpha-fimbriae, iron uptake systems (aerobactin, enterobactin, efeUOB) and curli adhesins. It is possible therefore that these factors are not sufficient for pathogenicity in the mammary gland, although they may possibly have an additive effect to pathogenicity when combined with the other factors found in MPEC.

### Phylogenetic analysis

**Fig 6:**
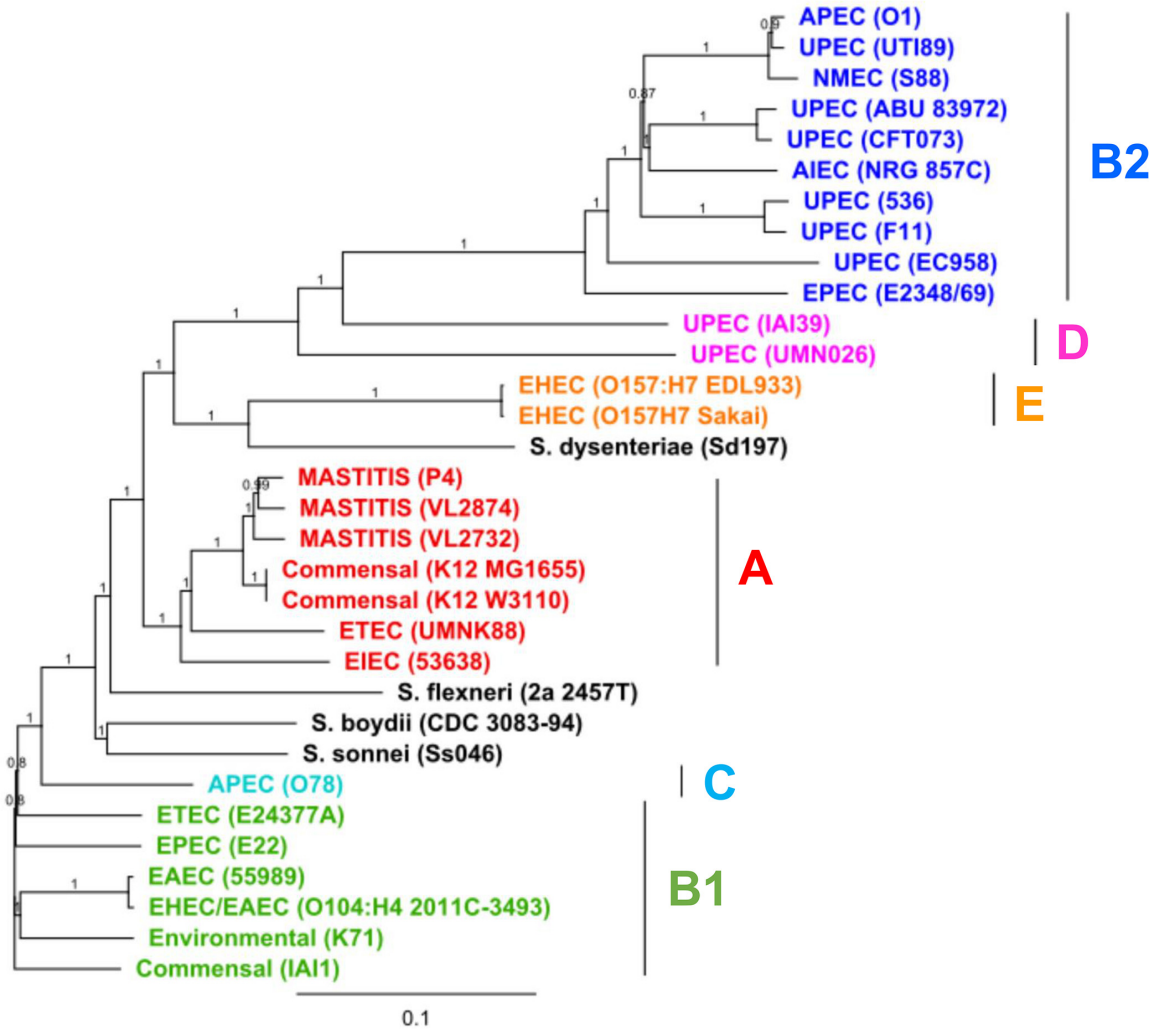
Whole genome SNP based phylogenetic analysis. Phylogenetic analysis of SNP extracted from whole genome alignment of the three MPEC strains (VL2874, VL2732 and P4), the environmental, non-mammary pathogenic strain K71 and representative strains of diverse *E*. *coli* pathotypes and non-pathogenic strains. Overall, the strains clustered according to their phylogenetic groups, indicated here by different colors. Confidence values are shown over each node.

Phylogenetic analysis was performed based on whole genome SNPs using the genome of strain K-12 MG1655 as a reference. All strains clustered according to their phylogenetic groups, as indicated in Fig 6. The MPEC strains studied here were therefore closely related to phylogenetic group A strains, while the environmental, non-mammary pathogenic strain K71 was closely related to phylogenetic group B1 strains, corroborating the phylogenetic assignment of these strains performed previously [18].

### Genome-to-Genome Distance (GGD)

**Fig 7:**
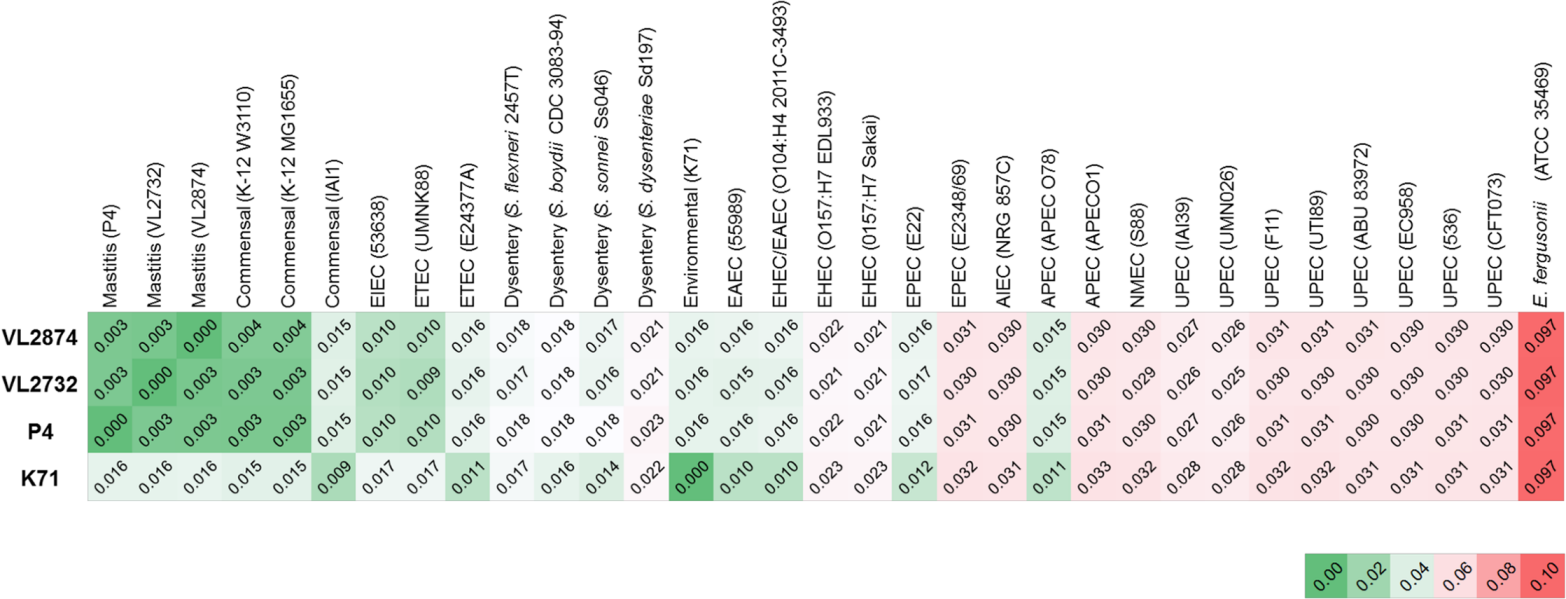
Genome-to-genome distance matrix. GGD was calculated between the four studied strains and various *E*. *coli* genomes from diverse pathotypes and non-pathogenic strains. The three mastitis pathogenic genomes were mostly similar, whereas notable genomic distances were found to other pathotypes and to the environmental, non-mammary pathogenic strain K71.

GGD was used to assess the overall similarity of genomes at the nucleotide sequences level. A heatmap of results is presented in Fig 7. Mastitis strains were most similar to each other compared to the other tested genomes. GGD between the three mastitis strains was 0.003, in contrast to 0.016 between mastitis strains and the non-MPEC strain K71. These values correspond to an estimated DDH value of 98% between mastitis strains, and 86% between mastitis strains and strain K71. Mastitis strains were also highly similar to the two strain K-12 variants genomes used in the comparison (MG1655 and W3110, GGD 0.003–4). The next highest similar genomes to the mastitis strains were EIEC (53638) and ETEC (UMNK88) (GGD 0.009–10) and in accord to the SNP based phylogeny. All other genomes in the set differed from the mastitis strains at the same level as strain K71 or more. Mastitis is an extra-intestinal infection, and thus it could be expected that mastitis strains would be similar to other extra-intestinal pathogenic *E*. *coli* (ExPEC). However, the most distant genomes in GGD were actually those of strains representing ExPEC, namely APEC, UPEC and NMEC, with estimated DDH values as low as 73%, which is close to the accepted cut-off for taxonomic differentiation at species level using classical DDH (70%). Overall, a similar range of DDH between *E*. *coli* genomes was previously reported [64]. Full detailed results including DNA-DNA hybridization (DDH) estimated values are provided in S3 Dataset.

### Proteome comparison

**Fig 8:**
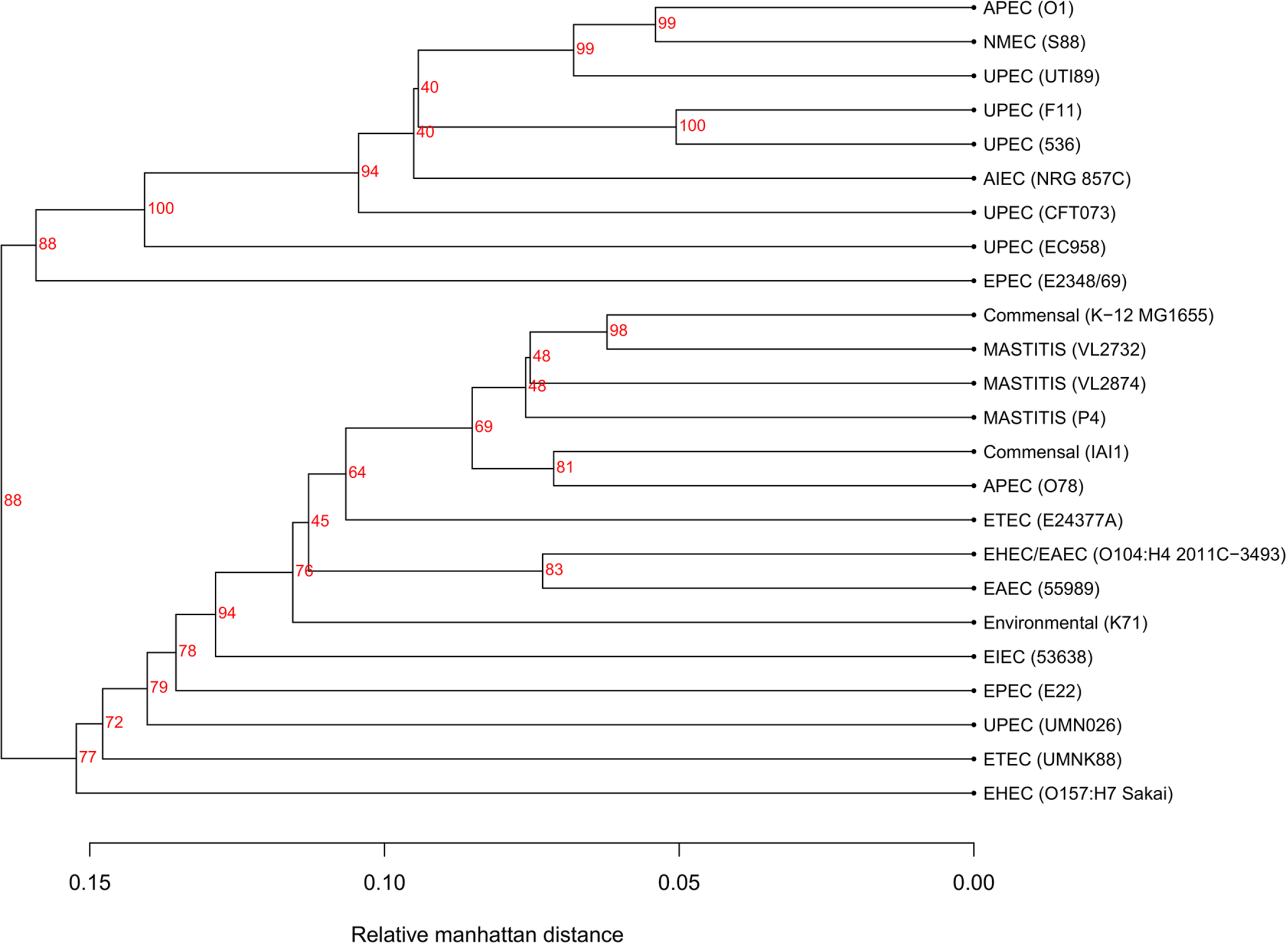
Relative Manhattan distance of predicted proteomes. Predicted proteomes of three mammary pathogenic *E*. *coli* and representatives of diverse *E*. *coli* pathotypes and non-pathogenic strains. Dendrogram generated on the basis of gene presence/absence considering all genes, including singletons, and validated after 100 bootstraps repetitions (depicted in red). Genes were clustered by 80% sequence identity over 80% sequence length. The three mammary pathogenic strains are closely related, and significantly distant from the environmental, non-mammary pathogenic strain K71.

Proteomes were predicted for representative genomes and compared pairwise for gene clustering into families. The Manhattan distance between the predicted proteomes based on gene family presence/absence is shown as a dendrogram in Fig 8. Overall, ExPEC, mainly UPEC genomes, clustered separately from most of the intestinal pathotypes. MPEC strains were included in the general intestinal *E*. *coli* cluster, and were separated from the non-mammary pathogenic strain K71. Similarity of mastitis strains and other *E*. *coli* proteomes ranged from 56% to 79%. A full similarity matrix of pairwise comparisons between proteomes is provided in S1 Fig. The similarity between MPEC strains and the non-mammary pathogenic strain K71 was 71.7%, 69.7% and 72.6% for strain VL2874, VL2732 and P4, respectively. In contrast, the similarity among mastitis strains was 80.5% between VL2874 and VL2732, 80.5% between VL2732 and P4, and 79.7% between VL2874 and P4.

Subsets of gene families were extracted for sequence-based comparison of predicted proteins between the three MPEC strains and strain K71. This analysis aimed to include predicted proteins not assigned a function during RAST annotation and thereby not included in the functional comparison above. The intersection of the three MPEC strains and excluding K71 sequences included 205 gene families. In addition, 271 (274 genes), 220 (221 genes) and 248 (251 genes) MPEC strain specific gene families were found in strains P4, VL2732 and VL2874, respectively. Annotation analysis of these gene families did not reveal the presence of genes not described above in the functional analysis. Most of sequences without a known annotation represented hypothetical proteins predicted by RAST. In addition, very few novel genes were found in the three MPEC strains. The intersection of the three MPEC strains and excluding all the other proteomes in the set comprised only eight gene families, five of which were hypothetical proteins. The three remaining families were not homologous to any predicted virulence factor. Whether any of these gene families have a role in the pathogenesis of MPEC is still to be studied. The gene families’ subsets are described in detail in S4 Dataset.

**Fig 9:**
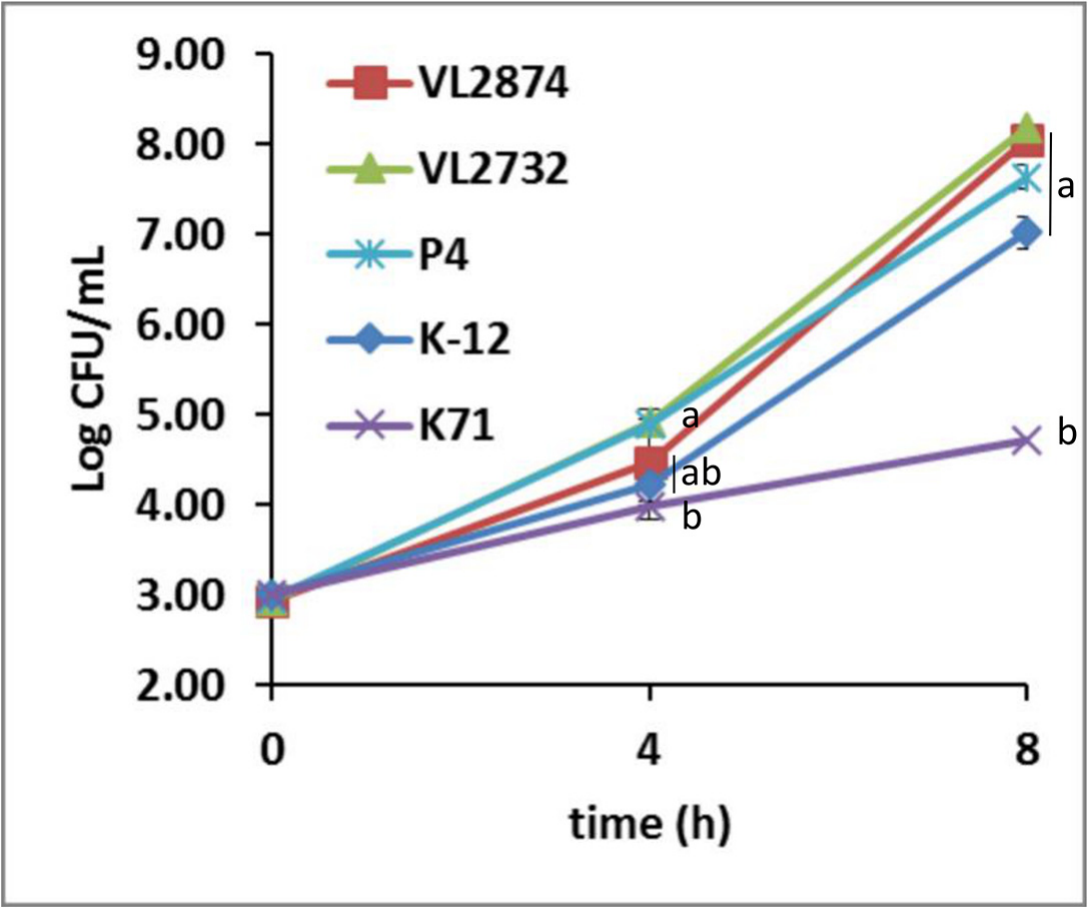
Growth rate in milk of MPEC (VL2874, VL2732 and P4), K-12 MG1655 and the environmental, non-mammary pathogenic strain K71. Error bars show SD of triplicate experiments. Statistically significant differences at the same time point are indicated by letters.

From the GGD and proteome analysis described above it is noteworthy that MPEC strains are closely related to strain K-12. The GGD results could be explained by the fact that the MPEC strains studied here share with K-12 the same genotypic background in terms of phylogenetic group (group A) and sequence type in MLST (ST10). The similarity between MPEC and K-12 was recently described also for other *E*. *coli* strains isolated from mastitis, based on phylogenetic analysis of conserved (core) genomic regions [65]. However, the proteome comparison presented here was based on presence/absence of genes considering all the genes found in the genomes, including non-conserved (accessory) regions, and is not necessarily in accord to phylogeny. The proteome comparison actually shows that the gene contents of the MPEC studied here are relatively close to that of K-12, suggesting two possible explanations. First, that only few genes in MPEC over the "basic" genes repertoire present in strain K-12 are necessary for pathogenicity in the mammary gland. Second, that the genome of K-12 may actually include genes promoting pathogenicity in the mammary gland. Due to the similarity between K-12 and MPEC, and since K-12 is widely considered a non-pathogenic strain, it was interesting to examine if K-12 could be pathogenic in the mammary gland. For this purpose, the growth in milk phenotype of K-12 was tested at first. As shown in Fig 9, K-12 is able to grow in milk in a rate similar to MPEC, and significantly different from K71, which consistently shows a slow growth rate in milk [21]. Growth in milk is a phenotype highly conserved in MPEC. As previously shown by Blum et al. [21], and later confirmed with a larger collection of MPEC and environmental strains from different farms (unpublished data), all MPEC strains are able to grow in milk at particularly high rates, whereas several *E*. *coli* strains present in the environment have slow growth rates in milk (like strain K71). Differences in growth rate were observed specifically in milk as all strains showed similar growth rates when bacteria were inoculated in regular nutrient broth (data not shown). Even though growth in milk is a phenotype of pivotal importance in *E*. *coli* pathogenicity in the mammary gland [6], this assay by itself does not confirm actual virulence potential in the gland. Thus, K-12 was also tested with the murine IMI model described above. In mice mammary glands, K-12 elicited a clear inflammatory response after 1 DPC, characterized by diffuse intra-alveolar neutrophil infiltration, but no observable inter-alveolar reaction or extensive damage to the gland tissue (Fig 1), and in fact a considerable number of milking alveoli was observed after challenge. No inflammation or tissue alterations were observed at 2 DPC, and milk producing alveoli remained conserved. K-12 bacteria were isolated from challenged mammary glands at 1 DPC. The inflammation elicited by K-12 was therefore somewhat milder than that of the MPEC strains studied here and that were actually isolated from mastitis. However, the potential of K-12 to cause mastitis cannot be discarded at this time. Hence it is possible that K-12 harbors genes that allow for pathogenicity in the mammary gland, partially explaining the close relatedness of MPEC and K-12 in the predicted proteome analysis.

The above observations on the pathogenicity of strain K-12 in the mammary gland were unexpected, given that this strain is largely regarded non-pathogenic, being often used as a negative control in pathogenicity assays, and its genome being used as a non-pathogenic reference for comparison in pathogenomic studies. However, the genome of strain K-12 encodes for various putative virulence factors. In fact, this strain was shown to be able to revert to a pathogenic phenotype under specific alterations in its transcriptional regulatory pathways [66] or upon restoration of O antigen biosynthesis [67]. The genome of strain K-12 includes some of the features listed above in common with the three MPEC genomes studied here and that are lacking in the non-mammary pathogenic strain K71. For instance, the galactosamine-specific IIB component, the sugar kinase cluster Ygc and the carbohydrate utilization cluster Ydj, the LPS enzymes 1,2-N-acetylglucosaminetransferase and LPS 1,6-galactosyltransferase and the LPS core biosynthesis protein RfaZ, the beta-fimbriae and the iron(III)-dicitrate system. In addition, similarly to the MPEC strains, strain K-12 has an intact D-serine cluster, it is able to metabolize D-serine and not sucrose, although it cannot metabolize uric acid [68]. The features described before found in each MPEC strain separately are not present in strain K-12. These features may therefore promote the increased pathogenicity observed with each MPEC strain studied.

### Summary

The comparison of mastitis strains representing different presentations of the disease with an experimentally-confirmed non-mammary pathogenic strain allowed the identification of common genes in MPEC that could be associated with pathogenicity in the mammary gland. It was also possible to identify MPEC strain-specific genes that may be associated with the different pathogenicity characteristics of each strain, and the different presentations of mastitis caused by each one. In addition, comparison of MPEC to a set of genomes representing other *E*. *coli* pathotypes showed high similarity between MPEC and divergence from other pathotypes at the whole genome and predicted proteome levels. The results presented here, notably the ability of strain K-12 to cause mild inflammation in the mammary gland and the very few novel genes found in MPEC genomes, suggest that minimal mammary pathogenicity may be associated with general metabolic, physiological, immunogenic or resistance features in *E*. *coli*, and not necessarily dependent on novel and unknown virulence factors specifically targeted at the mammary gland tissue. However, further MPEC genomes will need to be sequenced in order to identify the minimal genomic predictors characterizing the MPEC pathotype. This information would be valuable for the development of diagnostic tools aiming at MPEC specific identification and also the prediction of an MPEC isolate pathogenic potential, such as the likelihood of causing more acute or persistent mastitis. This would in turn allow for improved decision making in the management of an episode of *E*. *coli* mastitis, as in to treat, dry-off or cull affected animals, depending on the expected severity or persistency of disease. A comparative study of a larger number of MPEC genomes is currently underway.

Naturally, much attention is not given to non-mammary pathogenic strains from the dairy environment, and very few reports of such strains exist other than the present work [69]. Additional strains experimentally proven to be non-mammary pathogenic should be used in future comparative studies aiming at the identification of genes subject to positive selection in MPEC. Finally, functional studies either using specific genes' knockouts or gene expression experiments *in vivo* are needed to corroborate the role of the set of genes identified in MPEC here in the pathogenicity of mastitis.

## Supporting Information

S1 Dataset Results of phenotype array. (XLSX)

S2 Dataset RAST subsystems annotation. (XLSX)

S3 Dataset GGD analysis and list of genbank entries of genomes used for comparisons. (XLSX)

S4 Dataset BLAST2GO annotation of subsets of families of genes. (XLSX)

S1 Fig BLAST matrix of pairwise proteome comparisons. (PDF)

S1 Table Assembly summary. (PDF)

S2 Table Annotation summary. (PDF)

## References

[1] Halasa T, Nielen M, Huirne RBM, Hogeveen H (2009) Stochastic bio-economic model of bovine intramammary infection. Livest Sci 124: 295–305. Available: http://www.sciencedirect.com/science/article/pii/S1871141309000651. Accessed 12 May 2014.

[2] Hogeveen H, Huijps K, Lam TJGM (2011) Economic aspects of mastitis: new developments. N Z Vet J 59: 16–23. Available: http://www.ncbi.nlm.nih.gov/pubmed/21328153. Accessed 17 September 2014. 10.1080/00480169.2011.547165 21328153

[3] Le Maréchal C, Thiéry R, Vautor E, Le Loir Y (2011) Mastitis impact on technological properties of milk and quality of milk products-A review. Dairy Sci Technol 91: 247–282. Available: http://link.springer.com/10.1007/s13594-011-0009-6. Accessed 12 May 2014.

[4] Leitner G, Merin U, Silanikove N (2011) Effects of glandular bacterial infection and stage of lactation on milk clotting parameters: Comparison among cows, goats and sheep. Int Dairy J 21: 279–285. Available: http://www.sciencedirect.com/science/article/pii/S095869461000261X. Accessed 18 October 2014.

[5] Bradley AJ (2002) Bovine Mastitis: An Evolving Disease. Vet J 164: 116–128. Available: http://www.sciencedirect.com/science/article/pii/S1090023302907240. Accessed 3 June 2014. 12359466 10.1053/tvjl.2002.0724

[6] Hogan J, Smith KL (2003) Coliform mastitis. Vet Res 34: 507–519. Available: 10.1051/vetres:2003022. Accessed 16 June 2014. 14556693

[7] Blum SE, Heller ED, Leitner G (2014) Long term effects of Escherichia coli mastitis. Vet J 201: 72–77. Available: http://www.ncbi.nlm.nih.gov/pubmed/24906501. Accessed 22 April 2014. 10.1016/j.tvjl.2014.04.008 24906501

[8] Hill AW, Shears AL (1979) Recurrent coliform mastitis in the dairy cow. Vet Rec 105: 299–301. Available: http://www.ncbi.nlm.nih.gov/pubmed/390847. Accessed 2 December 2014. 390847 10.1136/vr.105.13.299

[9] Döpfer D, Barkema HW, Lam TJ, Schukken YH, Gaastra W (1999) Recurrent clinical mastitis caused by Escherichia coli in dairy cows. J Dairy Sci 82: 80–85. Available: http://www.journalofdairyscience.org/article/S0022030299752112/fulltext. Accessed 17 September 2014. 10022009 10.3168/jds.S0022-0302(99)75211-2

[10] Dogan B, Klaessig S, Rishniw M, Almeida RA, Oliver SP, et al (2006) Adherent and invasive Escherichia coli are associated with persistent bovine mastitis. Vet Microbiol 116: 270–282. Available: http://www.ncbi.nlm.nih.gov/pubmed/16787715. Accessed 22 April 2014. 16787715 10.1016/j.vetmic.2006.04.023

[11] Almeida RA, Dogan B, Klaessing S, Schukken YH, Oliver SP (2011) Intracellular fate of strains of Escherichia coli isolated from dairy cows with acute or chronic mastitis. Vet Res Commun 35: 89–101. Available: http://www.ncbi.nlm.nih.gov/pubmed/21207146. Accessed 17 September 2014. 10.1007/s11259-010-9455-5 21207146

[12] Kaper JB, Nataro JP, Mobley HL (2004) Pathogenic Escherichia coli. Nat Rev Microbiol 2: 123–140. Available: http://www.ncbi.nlm.nih.gov/pubmed/15040260. Accessed 21 March 2014. 15040260 10.1038/nrmicro818

[13] Shpigel NY, Elazar S, Rosenshine I (2008) Mammary pathogenic Escherichia coli. Curr Opin Microbiol 11: 60–65. Available: http://www.sciencedirect.com/science/article/pii/S1369527408000052. Accessed 18 September 2014. 10.1016/j.mib.2008.01.004 18291708

[14] Porcherie A, Cunha P, Trotereau A, Roussel P, Gilbert FB, et al (2012) Repertoire of Escherichia coli agonists sensed by innate immunity receptors of the bovine udder and mammary epithelial cells. Vet Res 43: 14 Available: http://www.veterinaryresearch.org/content/43/1/14. Accessed 6 April 2014. 10.1186/1297-9716-43-14 22330199 PMC3305352

[15] Wenz JRR, Barrington GMM, Garry FBB, Ellis RPP, Magnuson RJJ (2006) Escherichia coli isolates’ serotypes, genotypes, and virulence genes and clinical coliform mastitis severity. J Dairy Sci 89: 3408–3412. Available: http://www.journalofdairyscience.org/article/S0022030206723773/fulltext. Accessed 17 September 2014. 16899673 10.3168/jds.S0022-0302(06)72377-3

[16] Liu Y, Liu G, Liu W, Liu Y, Ali T, et al (2014) Phylogenetic group, virulence factors and antimicrobial resistance of Escherichia coli associated with bovine mastitis. Res Microbiol 165: 273–277. Available: http://www.ncbi.nlm.nih.gov/pubmed/24705087. Accessed 19 August 2014. 10.1016/j.resmic.2014.03.007 24705087

[17] Fernandes JBC, Zanardo LG, Galvão NN, Carvalho IA, Nero LA, et al (2011) Escherichia coli from clinical mastitis: serotypes and virulence factors. J Vet Diagn Invest 23: 1146–1152. Available: http://www.ncbi.nlm.nih.gov/pubmed/22362795. Accessed 17 September 2014. 10.1177/1040638711425581 22362795

[18] Blum SE, Leitner G (2013) Genotyping and virulence factors assessment of bovine mastitis Escherichia coli. Vet Microbiol 163: 305–312. Available: http://www.ncbi.nlm.nih.gov/pubmed/23374653. Accessed 19 March 2014. 10.1016/j.vetmic.2012.12.037 23374653

[19] Suojala L, Pohjanvirta T, Simojoki H, Myllyniemi A-L, Pitkälä A, et al (2011) Phylogeny, virulence factors and antimicrobial susceptibility of Escherichia coli isolated in clinical bovine mastitis. Vet Microbiol 147: 383–388. Available: http://www.sciencedirect.com/science/article/pii/S0378113510003469. Accessed 2 May 2014. 10.1016/j.vetmic.2010.07.011 20729012

[20] Dogan B, Rishniw M, Bruant G, Harel J, Schukken YH, et al (2012) Phylogroup and lpfA influence epithelial invasion by mastitis associated Escherichia coli. Vet Microbiol 159: 163–170. Available: http://www.sciencedirect.com/science/article/pii/S0378113512002003. Accessed 3 September 2014. 10.1016/j.vetmic.2012.03.033 22510704

[21] Blum SE, Heller ED, Krifucks O, Sela S, Hammer-Muntz O, et al (2008) Identification of a bovine mastitis Escherichia coli subset. Vet Microbiol 132: 135–148. Available: http://www.ncbi.nlm.nih.gov/pubmed/18571344. Accessed 19 March 2014. 10.1016/j.vetmic.2008.05.012 18571344

[22] Srinivasan V, Gillespie BE, Lewis MJ, Nguyen LT, Headrick SI, et al (2007) Phenotypic and genotypic antimicrobial resistance patterns of Escherichia coli isolated from dairy cows with mastitis. Vet Microbiol 124: 319–328. Available: http://www.sciencedirect.com/science/article/pii/S0378113507002222. Accessed 19 April 2015. 17544234 10.1016/j.vetmic.2007.04.040

[23] Döpfer D, Almeida RA, Lam TJG, Nederbragt H, Oliver S, et al (2000) Adhesion and invasion of Escherichia coli from single and recurrent clinical cases of bovine mastitis in vitro. Vet Microbiol 74: 331–343. Available: http://www.sciencedirect.com/science/article/pii/S0378113500001917. Accessed 17 September 2014. 10831855 10.1016/s0378-1135(00)00191-7

[24] Kerro Dego O, Oliver SP, Almeida RA (2012) Host-pathogen gene expression profiles during infection of primary bovine mammary epithelial cells with Escherichia coli strains associated with acute or persistent bovine mastitis. Vet Microbiol 155: 291–297. Available: http://www.sciencedirect.com/science/article/pii/S0378113511004627. Accessed 17 September 2014. 10.1016/j.vetmic.2011.08.016 21917386

[25] Döpfer D, Nederbragt H, Almeida RA, Gaastra W (2001) Studies about the mechanism of internalization by mammary epithelial cells of Escherichia coli isolated from persistent bovine mastitis. Vet Microbiol 80: 285–296. Available: http://www.sciencedirect.com/science/article/pii/S0378113501003078. Accessed 17 September 2014. 11337144 10.1016/s0378-1135(01)00307-8

[26] Bramley A (1976) Variations in the susceptibility of lactating and non-lactating bovine udders to infection when infused with Escherichia coli. J Dairy Sci 43: 205–211. 10.1017/s0022029900015752 783219

[27] Blum SE, Sela N, Heller ED, Sela S, Leitner G (2012) Genome analysis of bovine-mastitis-associated Escherichia coli O32:H37 strain P4. J Bacteriol 194: 3732 Available: http://www.pubmedcentral.nih.gov/articlerender.fcgi?artid=3393488&tool=pmcentrez&rendertype=abstract. Accessed 19 March 2014. 10.1128/JB.00535-12 22740662 PMC3393488

[28] Seroussi E, Klompus S, Silanikove M, Krifucks O, Shapiro F, et al (2013) Nonbactericidal secreted phospholipase A2s are potential anti-inflammatory factors in the mammary gland. Immunogenetics 65: 861–871. Available: http://www.ncbi.nlm.nih.gov/pubmed/24091988. Accessed 2 September 2014. 10.1007/s00251-013-0738-1 24091988

[29] Bochner BR (2009) Global phenotypic characterization of bacteria. FEMS Microbiol Rev 33: 191–205. Available: http://www.pubmedcentral.nih.gov/articlerender.fcgi?artid=2704929&tool=pmcentrez&rendertype=abstract. Accessed 1 September 2014. 10.1111/j.1574-6976.2008.00149.x 19054113 PMC2704929

[30] R Development Core Team (2014) R: A language and environment for statistical computing. Available: http://www.r-project.org/.

[31] Vaas LA, Sikorski J, Hofner B, Fiebig A, Buddruhs N, et al (2013) opm: an R package for analysing OmniLog(R) phenotype microarray data. Bioinformatics 29: 1823–1824. Available: http://www.ncbi.nlm.nih.gov/pubmed/23740744. Accessed 6 October 2014. 10.1093/bioinformatics/btt291 23740744

[32] Vaas LA, Sikorski J, Michael V, Göker M, Klenk H-P (2012) Visualization and curve-parameter estimation strategies for efficient exploration of phenotype microarray kinetics. PLoS One 7: e34846 Available: http://www.pubmedcentral.nih.gov/articlerender.fcgi?artid=3334903&tool=pmcentrez&rendertype=abstract. Accessed 24 September 2014. 10.1371/journal.pone.0034846 22536335 PMC3334903

[33] Kado CI, Liu ST (1981) Rapid procedure for detection and isolation of large and small plasmids. J Bacteriol 145: 1365–1373. Available: http://www.pubmedcentral.nih.gov/articlerender.fcgi?artid=217141&tool=pmcentrez&rendertype=abstract. Accessed 4 June 2015. 7009583 10.1128/jb.145.3.1365-1373.1981 PMC217141

[34] Darling AE, Mau B, Perna NT (2010) progressiveMauve: multiple genome alignment with gene gain, loss and rearrangement. PLOS ONE 5: e11147 Available: http://www.pubmedcentral.nih.gov/articlerender.fcgi?artid=2892488&tool=pmcentrez&rendertype=abstract. Accessed 19 March 2014. 10.1371/journal.pone.0011147 20593022 PMC2892488

[35] Carver TJ, Rutherford KM, Berriman M, Rajandream M-A, Barrell BG, et al (2005) ACT: the Artemis Comparison Tool. Bioinformatics 21: 3422–3423. Available: http://europepmc.org/abstract/MED/15976072. Accessed 23 July 2014. 15976072 10.1093/bioinformatics/bti553

[36] Rutherford K, Parkhill J, Crook J, Horsnell T, Rice P, et al (2000) Artemis: sequence visualization and annotation. Bioinformatics 16: 944–945. Available: http://europepmc.org/abstract/MED/11120685. Accessed 17 August 2014. 11120685 10.1093/bioinformatics/16.10.944

[37] Aziz RK, Bartels D, Best AA, DeJongh M, Disz T, et al (2008) The RAST Server: rapid annotations using subsystems technology. BMC Genomics 9: 75 Available: http://www.pubmedcentral.nih.gov/articlerender.fcgi?artid=2265698&tool=pmcentrez&rendertype=abstract. Accessed 19 March 2014. 10.1186/1471-2164-9-75 18261238 PMC2265698

[38] Zhang Z, Schwartz S, Wagner L, Miller W (2000) A greedy algorithm for aligning DNA sequences. J Comput Biol 7: 203–214. Available: http://www.ncbi.nlm.nih.gov/pubmed/10890397. Accessed 28 August 2014. 10890397 10.1089/10665270050081478

[39] Carattoli A, Zankari E, García-Fernández A, Voldby Larsen M, Lund O, et al (2014) In Silico Detection and Typing of Plasmids using PlasmidFinder and Plasmid Multilocus Sequence Typing. Antimicrob Agents Chemother 58: 3895–3903. Available: http://www.pubmedcentral.nih.gov/articlerender.fcgi?artid=4068535&tool=pmcentrez&rendertype=abstract. Accessed 14 July 2014. 10.1128/AAC.02412-14 24777092 PMC4068535

[40] Leekitcharoenphon P, Kaas RS, Thomsen MCF, Friis C, Rasmussen S, et al (2012) snpTree—a web-server to identify and construct SNP trees from whole genome sequence data. BMC Genomics 13 Suppl 7: S6 Available: http://www.biomedcentral.com/1471-2164/13/S7/S6. Accessed 19 June 2015. 10.1186/1471-2164-13-S7-S6 23281601 PMC3521233

[41] Price MN, Dehal PS, Arkin AP (2010) FastTree 2—approximately maximum-likelihood trees for large alignments. PLoS One 5: e9490 Available: http://journals.plos.org/plosone/article?id=10.1371/journal.pone.0009490. Accessed 10 July 2014. 10.1371/journal.pone.0009490 20224823 PMC2835736

[42] Meier-Kolthoff JP, Auch AF, Klenk H-P, Göker M (2013) Genome sequence-based species delimitation with confidence intervals and improved distance functions. BMC Bioinformatics 14: 60 Available: http://www.biomedcentral.com/1471-2105/14/60. Accessed 2 September 2014. 10.1186/1471-2105-14-60 23432962 PMC3665452

[43] Vesth T, Lagesen K, Acar Ö, Ussery DW (2013) CMG-biotools, a free workbench for basic comparative microbial genomics. PLOS ONE 8: e60120 Available: http://www.pubmedcentral.nih.gov/articlerender.fcgi?artid=3618517&tool=pmcentrez&rendertype=abstract. Accessed 19 March 2014. 10.1371/journal.pone.0060120 23577086 PMC3618517

[44] Wattam AR, Abraham D, Dalay O, Disz TL, Driscoll T, et al (2014) PATRIC, the bacterial bioinformatics database and analysis resource. Nucleic Acids Res 42: D581–D591. Available: http://nar.oxfordjournals.org/content/early/2013/11/12/nar.gkt1099.full. Accessed 14 August 2014. 10.1093/nar/gkt1099 24225323 PMC3965095

[45] Altschul SF, Gish W, Miller W, Myers EW, Lipman DJ (1990) Basic local alignment search tool. J Mol Biol 215: 403–410. Available: http://www.ncbi.nlm.nih.gov/pubmed/2231712. Accessed 19 March 2014. 2231712 10.1016/S0022-2836(05)80360-2

[46] Conesa A, Götz S, García-Gómez JM, Terol J, Talón M, et al (2005) Blast2GO: a universal tool for annotation, visualization and analysis in functional genomics research. Bioinformatics 21: 3674–3676. Available: http://bioinformatics.oxfordjournals.org/content/21/18/3674.full. Accessed 9 July 2014. 16081474 10.1093/bioinformatics/bti610

[47] Notebaert S, Meyer E (2006) Mouse models to study the pathogenesis and control of bovine mastitis. A review. Vet Q 28: 2–13. Available: http://www.ncbi.nlm.nih.gov/pubmed/16605156. Accessed 2 December 2014. 16605156 10.1080/01652176.2006.9695201

[48] Chandler RL, Anger HS (1977) Ultrastructural and associated studies on experimental mastitis in the mouse produced by Escherichia coli and other bacterial species. J Comp Pathol 87: 471–485. 409741 10.1016/0021-9975(77)90036-6

[49] Moritz RL, Welch RA (2006) The Escherichia coli argW-dsdCXA genetic island is highly variable, and E. coli K1 strains commonly possess two copies of dsdCXA. J Clin Microbiol 44: 4038–4048. 10.1128/JCM.01172-06 17088369 PMC1698345

[50] Connolly JP, Goldstone RJ, Burgess K, Cogdell RJ, Beatson SA, et al (2014) The host metabolite D-serine contributes to bacterial niche specificity through gene selection. ISME J: 1–13. Available: http://www.nature.com/doifinder/10.1038/ismej.2014.242. Accessed 21 August 2015.

[51] Silanikove N, Shapiro F, Leitner G (2007) Posttranslational ruling of xanthine oxidase activity in bovine milk by its substrates. Biochem Biophys Res Commun 363: 561–565. Available: http://www.sciencedirect.com/science/article/pii/S0006291X07019390. Accessed 9 November 2014. 17888877 10.1016/j.bbrc.2007.08.188

[52] Burvenich C, Van Merris V, Mehrzad J, Diez-Fraile A, Duchateau L (2003) Severity of E. coli mastitis is mainly determined by cow factors. Vet Res 34: 521–564. Available: 10.1051/vetres:2003023. Accessed 15 September 2014. 14556694

[53] Lin J, Hogan JS, Smith KL (1999) Antigenic Homology of the Inducible Ferric Citrate Receptor (FecA) of Coliform Bacteria Isolated from Herds with Naturally Occurring Bovine Intramammary Infections. Clin Diagn Lab Immunol 6: 966–969. Available: http://cvi.asm.org/content/6/6/966.long. Accessed 15 September 2014. 10548594 10.1128/cdli.6.6.966-969.1999 PMC95806

[54] Leimbach A, Poehlein A, Witten A, Scheutz F, Schukken Y, et al (2015) Complete Genome Sequences of Escherichia coli Strains 1303 and ECC-1470 Isolated from Bovine Mastitis. Genome Announc 3: e00182–15. Available: http://genomea.asm.org/content/3/2/e00182-15.full. Accessed 5 June 2015. 10.1128/genomeA.00182-15 25814601 PMC4384141

[55] Vigil PD, Alteri CJ, Mobley HLT (2011) Identification of in vivo-induced antigens including an RTX family exoprotein required for uropathogenic Escherichia coli virulence. Infect Immun 79: 2335–2344. Available: http://iai.asm.org/content/79/6/2335.long. Accessed 14 September 2014. 10.1128/IAI.00110-11 21422188 PMC3125824

[56] Chen C-Y, Nace GW, Solow B, Fratamico P (2007) Complete nucleotide sequences of 84.5- and 3.2-kb plasmids in the multi-antibiotic resistant Salmonella enterica serovar Typhimurium U302 strain G8430. Plasmid 57: 29–43. Available: http://www.sciencedirect.com/science/article/pii/S0147619X06000564. Accessed 23 June 2015. 16828159 10.1016/j.plasmid.2006.05.005

[57] Wang X, Wood TK (2011) Toxin-antitoxin systems influence biofilm and persister cell formation and the general stress response. Appl Environ Microbiol 77: 5577–5583. Available: http://aem.asm.org/content/77/16/5577.long. Accessed 31 July 2014. 10.1128/AEM.05068-11 21685157 PMC3165247

[58] Hancock V, Ferrières L, Klemm P (2008) The ferric yersiniabactin uptake receptor FyuA is required for efficient biofilm formation by urinary tract infectious Escherichia coli in human urine. Microbiology 154: 167–175. Available: http://mic.sgmjournals.org/content/154/1/167.full. Accessed 14 September 2014. 10.1099/mic.0.2007/011981-0 18174135

[59] Chaturvedi KS, Hung CS, Giblin DE, Urushidani S, Austin AM, et al (2014) Cupric yersiniabactin is a virulence-associated superoxide dismutase mimic. ACS Chem Biol 9: 551–561. Available: 10.1021/cb400658k. Accessed 14 September 2014. 10.1021/cb400658k 24283977 PMC3934373

[60] Huja S, Oren Y, Trost E, Brzuszkiewicz E, Biran D, et al (2015) Genomic avenue to avian colisepticemia. MBio 6 Available: http://www.pubmedcentral.nih.gov/articlerender.fcgi?artid=4313913&tool=pmcentrez&rendertype=abstract. Accessed 19 June 2015. 10.1128/mBio.01681-14 PMC4313913 25587010

[61] Schubert S, Rakin A, Heesemann J (2004) The Yersinia high-pathogenicity island (HPI): evolutionary and functional aspects. Int J Med Microbiol 294: 83–94. Available: http://www.ncbi.nlm.nih.gov/pubmed/15493818. Accessed 14 September 2014. 10.1016/j.ijmm.2004.06.026 15493818

[62] Sabri M, Léveillé S, Dozois CM (2006) A SitABCD homologue from an avian pathogenic Escherichia coli strain mediates transport of iron and manganese and resistance to hydrogen peroxide. Microbiology 152: 745–758. Available: http://www.ncbi.nlm.nih.gov/pubmed/16514154. Accessed 14 September 2014. 16514154 10.1099/mic.0.28682-0

[63] White LJ, Schukken YH, Dogan B, Green L, Döpfer D, et al (2010) Modelling the dynamics of intramammary E. coli infections in dairy cows: understanding mechanisms that distinguish transient from persistent infections. Vet Res 41: 13 Available: http://www.pubmedcentral.nih.gov/articlerender.fcgi?artid=2789329&tool=pmcentrez&rendertype=abstract. Accessed 15 January 2015. 10.1051/vetres/2009061 19840536 PMC2789329

[64] Goris J, Konstantinidis KT, Klappenbach JA, Coenye T, Vandamme P, et al (2007) DNA-DNA hybridization values and their relationship to whole-genome sequence similarities. Int J Syst Evol Microbiol 57: 81–91. Available: http://www.ncbi.nlm.nih.gov/pubmed/17220447. Accessed 19 August 2014. 17220447 10.1099/ijs.0.64483-0

[65] Richards VP, Lefébure T, Pavinski Bitar PD, Dogan B, Simpson KW, et al (2015) Genome Based Phylogeny and Comparative Genomic Analysis of Intra-Mammary Pathogenic Escherichia coli. PLOS ONE 10: e0119799 Available: http://www.pubmedcentral.nih.gov/articlerender.fcgi?artid=4373696&tool=pmcentrez&rendertype=abstract. Accessed 30 March 2015. 10.1371/journal.pone.0119799 25807497 PMC4373696

[66] Koli P, Sudan S, Fitzgerald D, Adhya S, Kar S (2011) Conversion of Commensal Escherichia coli K-12 to an Invasive Form via Expression of a Mutant Histone-Like Protein. MBio 2: e00182–11 –e00182–11. Available: http://www.pubmedcentral.nih.gov/articlerender.fcgi?artid=3172693&tool=pmcentrez&rendertype=abstract. Accessed 15 March 2015. 10.1128/mBio.00182-11 PMC3172693 21896677

[67] Browning DF, Wells TJ, França FLS, Morris FC, Sevastsyanovich YR, et al (2013) Laboratory adapted Escherichia coli K-12 becomes a pathogen of Caenorhabditis elegans upon restoration of O antigen biosynthesis. Mol Microbiol 87: 939–950. 10.1111/mmi.12144 23350972

[68] Keseler IM, Mackie A, Peralta-Gil M, Santos-Zavaleta A, Gama-Castro S, et al (2013) EcoCyc: fusing model organism databases with systems biology. Nucleic Acids Res 41: D605–D612. Available: http://nar.oxfordjournals.org/content/41/D1/D605.long. Accessed 23 February 2015. 10.1093/nar/gks1027 23143106 PMC3531154

[69] Anderson JC, Burrows MR, Bramley AJ (1977) Bacterial Adherence in Mastitis Caused by Escherichia coli. 14: 618–628. 10.1177/030098587701400608 337635

