# Supplementary figures and images for: Genomic and Phenomic Study of Mammary Pathogenic Escherichia coli
Source: PLoS One. 2015 Sep 1;10(9):e0136387. doi: 10.1371/journal.pone.0136387 (PMC4556653; doi:10.1371/journal.pone.0136387)

Homology between proteomes

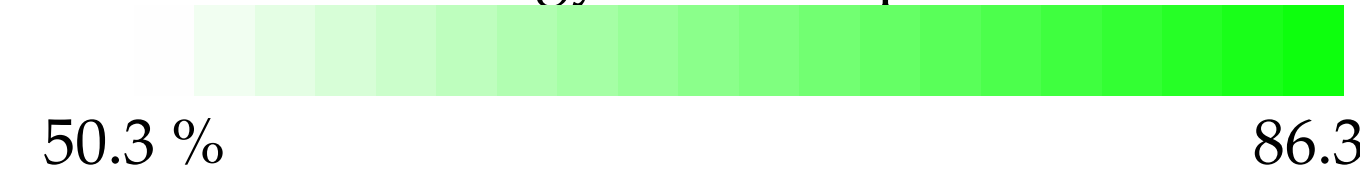

Homology within proteomes

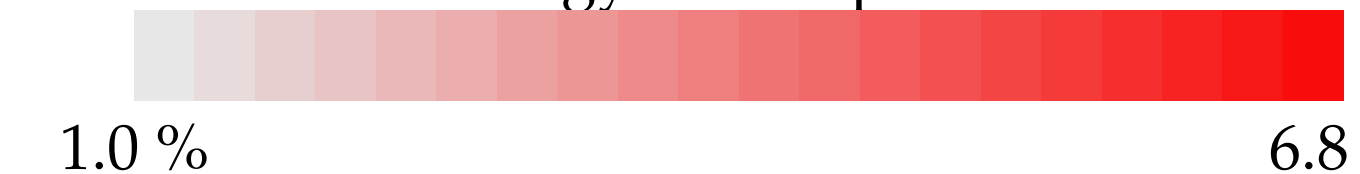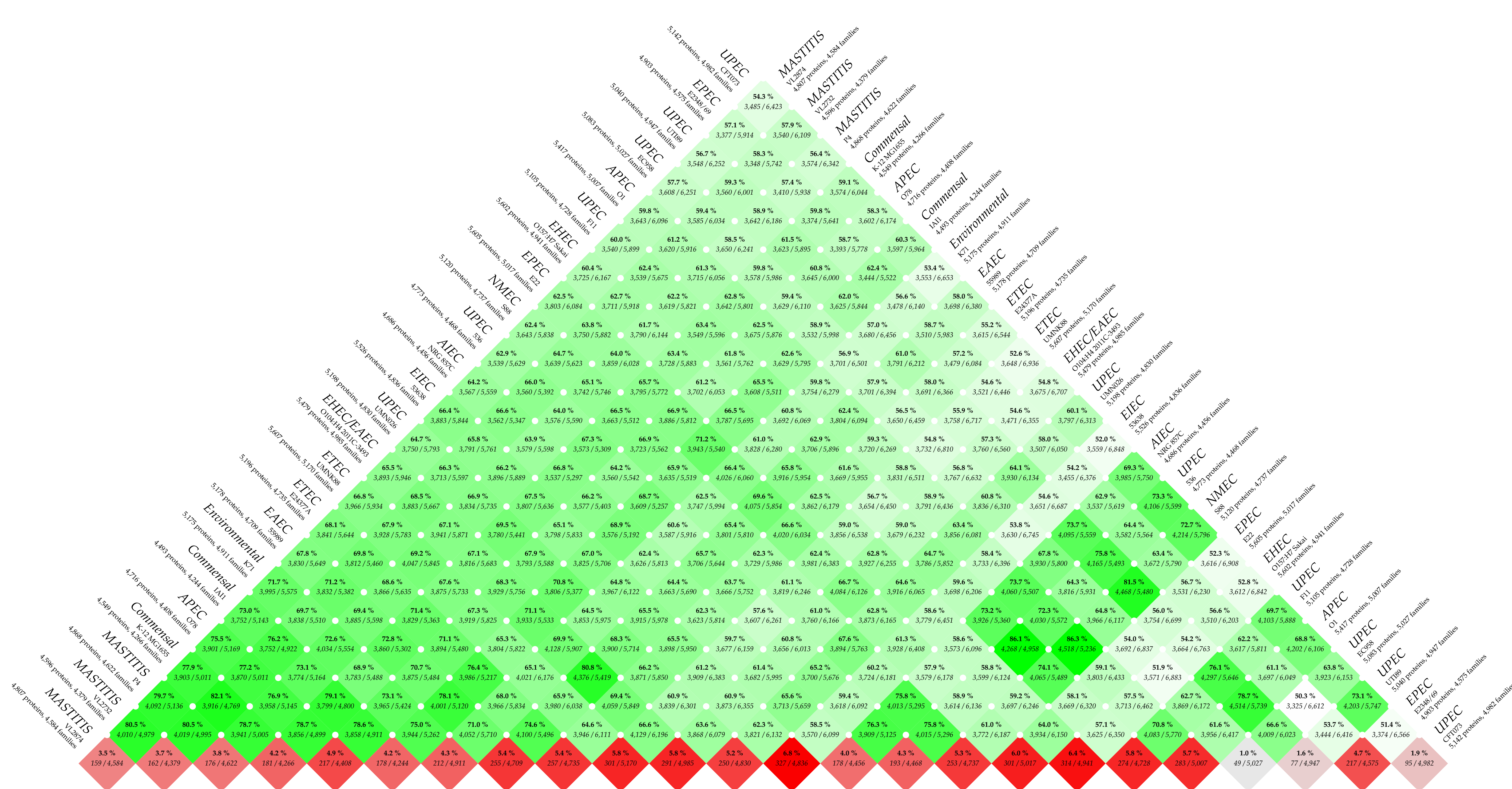

Supplement: S1 Fig — (PDF) [file pone.0136387.s005.pdf]
