# Supplementary material for: Genomic and Phenomic Study of Mammary Pathogenic Escherichia coli
Source: PLoS One. 2015 Sep 1;10(9):e0136387. doi: 10.1371/journal.pone.0136387 (PMC4556653; doi:10.1371/journal.pone.0136387)
Supplement: S1 Table — (PDF) [file pone.0136387.s006.pdf]

S2 Table *De novo* assembly metrics of the bacterial genomes studied.

Strain	No. contigs (> 500bp)	N50	Largest contig length	Mean read depth (std)	Inferred read error %	Q40-plus bases %
**VL2874**	141 (111)	88833	229894	25 (26)	1.17	99.41
**VL2732**	91 (69)	116446	410781	35 (30)	1.03	99.80
**P4**	87 (63)	165669	459864	35 (32)	1.02	99.77
**K71**	120 (94)	108569	334644	29 (36)	1.17	99.68
