# Supplementary material for: Genomic and Phenomic Study of Mammary Pathogenic Escherichia coli
Source: PLoS One. 2015 Sep 1;10(9):e0136387. doi: 10.1371/journal.pone.0136387 (PMC4556653; doi:10.1371/journal.pone.0136387)
Supplement: S2 Table — (PDF) [file pone.0136387.s007.pdf]

S3 Table. Statistics of RAST annotation results.

Strain	Genome size(bp)	%GC	Contigs with PEGs	No. subsystems	Coding sequences	rRNA	tRNA	%SEED subsystem coverage	Hypothetical (%)
**VL2874**	4786508	51	141	587	4807	5	71	60	1079 (22)
**VL2732**	4665867	51	91	585	4596	5	74	60	1010 (22)
**P4**	4874634	51	87	587	4868	11	71	59	1106 (23)
**K71**	5117165	51	120	598	5175	4	68	58	1245 (24)
